# Supplementary material for: Cyclometalated and NNN Terpyridine Ruthenium Photocatalysts and Their Cytotoxic Activity
Source: Molecules. 2024 May 5;29(9):2146. doi: 10.3390/molecules29092146 (PMC11085208; doi:10.3390/molecules29092146)
Supplement: Supplementary file 1 [file molecules-29-02146-s001.zip › molecules-2965495-supplementary.pdf]

Molecules

Cyclometalated and NNN Terpyridine Ruthenium Photocatalysts and their Cytotoxic Activity

Maurizio Ballico[1,*], Dario Alessi[1], Eleonora Aneggi[1], Marta Busato[1], Daniele Zuccaccia[1], Lorenzo Allegri[2], Giuseppe Damante[2], Christian Jandl[3] and Walter Baratta[1,*]

[1]Dipartimento di Scienze Agroalimentari, Ambientali e Animali, Università di Udine, Via Cotonificio 108, I-33100 Udine, Italy; alessi.dario@spes.uniud.it (D.A.); eleonora.aneggi@uniud.it (E.A.); marta.busato@uniud.it (M.B.); daniele.zuccaccia@uniud.it (D.Z.) [2]Dipartimento di Area Medica – Istituto di Genetica Medica, Università di Udine, Via Chiusaforte, F3, I-33100 Udine, Italy; allegri.lorenzo@spes.uniud.it (L.A.); giuseppe.damante@uniud.it (G.D.) [3]Department of Chemistry & Catalysis Research Center, Technische Universität München, Ernst-Otto-Fischer-Str. 1, 85748 Garching b. München, Germany; christian.jandl@tum.de (C.J.)

* Correspondence: maurizio.ballico@uniud.it; walter.baratta@uniud.it

Supporting Information

Table of Contents:

Figure S1. $^{31}P\{^1H\}$ NMR spectrum of [Ru(η^2-OAc)(NC-tpy)(dppb)] (**1**) Pag. S5

Figure S2. ^1H NMR spectrum of [Ru(η^2-OAc)(NC-tpy)(dppb)] (**1**) Pag. S6

Figure S3. $^{13}C\{^1H\}$ DEPTQ NMR spectrum of [Ru(η^2-OAc)(NC-tpy)(dppb)] (**1**) Pag. S7

Figure S4. ^1H-^1H COSY 2D NMR spectrum of [Ru(η^2-OAc)(NC-tpy)(dppb)] (**1**) Pag. S8

Figure S5. ^1H-^{13}C HSQC 2D NMR spectrum of [Ru(η^2-OAc)(NC-tpy)(dppb)] (**1**) Pag. S9

Figure S6. ^1H-^{13}C HMBC 2D NMR spectrum of [Ru(η^2-OAc)(NC-tpy)(dppb)] (**1**) Pag. S10

Figure S7. ^1H-^{31}P HMBC 2D NMR spectrum of [Ru(η^2-OAc)(NC-tpy)(dppb)] (**1**) Pag. S11

Figure S8. ^1H-^1H NOESY 2D NMR spectrum of [Ru(η^2-OAc)(NC-tpy)(dppb)] (**1**) Pag. S12

Figure S9. $^{31}P\{^1H\}$ NMR spectrum of [Ru(η^2-OAc)$_2$((*R*,*R*)-Skewphos)] (**2**) Pag. S13

Figure S10. ^1H NMR spectrum of [Ru(η^2-OAc)$_2$((*R*,*R*)-Skewphos)] (**2**) Pag. S14

Figure S11. ^{13}C{^{1}H} DEPTQ NMR spectrum of [Ru(η^{2}-OAc)$_{2}$((*R*,*R*)-Skewphos)] (**2**) Pag. S15

Figure S12. ^{1}H-^{1}H COSY 2D NMR spectrum of [Ru(η^{2}-OAc)$_{2}$((*R*,*R*)-Skewphos)] (**2**) Pag. S16

Figure S13. ^{1}H-^{13}C HSQC 2D NMR spectrum of [Ru(η^{2}-OAc)$_{2}$((*R*,*R*)-Skewphos)] (**2**) Pag. S17

Figure S14. ^{1}H-^{31}P HMBC 2D NMR spectrum of [Ru(η^{2}-OAc)$_{2}$((*R*,*R*)-Skewphos)] (**2**) Pag. S18

Figure S15. ^{1}H-^{1}H NOESY 2D NMR spectrum of [Ru(η^{2}-OAc)$_{2}$((*R*,*R*)-Skewphos)] (**2**) Pag. S19

Figure S16. ^{31}P{^{1}H} NMR spectrum of [Ru(η^{2}-OAc)(NC-tpy)((*R*,*R*)-Skewphos)] (**4**) Pag. S20

Figure S17. ^{1}H NMR spectrum of [Ru(η^{2}-OAc)(NC-tpy)((*R*,*R*)-Skewphos)] (**4**) Pag. S21

Figure S18. ^{13}C{^{1}H} DEPTQ NMR spectrum of [Ru(η^{2}-OAc)(NC-tpy)((*R*,*R*)-Skewphos)] (**4**)

Pag. S22

Figure S19. ^{1}H-^{1}H COSY 2D NMR spectrum of [Ru(η^{2}-OAc)(NC-tpy)((*R*,*R*)-Skewphos)] (**4**)

Pag. S23

Figure S20. ^{1}H-^{13}C HSQC 2D NMR spectrum of [Ru(η^{2}-OAc)(NC-tpy)((*R*,*R*)-Skewphos)] (**4**)

Pag. S24

Figure S21. ^{1}H-^{13}C HMBC 2D NMR spectrum of [Ru(η^{2}-OAc)(NC-tpy)((*R*,*R*)-Skewphos)] (**4**)

Pag. S25

Figure S22. ^{1}H-^{31}P HMBC 2D NMR spectrum of [Ru(η^{2}-OAc)(NC-tpy)((*R*,*R*)-Skewphos)] (**4**)

Pag. S26

Figure S23. ^{1}H-^{1}H NOESY 2D NMR spectrum of [Ru(η^{2}-OAc)(NC-tpy)((*R*,*R*)-Skewphos)] (**4**)

Pag. S27

Figure S24. ^{31}P{^{1}H} NMR spectrum of [Ru(η^{1}-OAc)(NNN-tpy)((*R*,*R*)-Skewphos)]OAc (**6**)

Pag. S28

Figure S25. ^{1}H NMR spectrum of [Ru(η^{1}-OAc)(NNN-tpy)((*R*,*R*)-Skewphos)]OAc (**6**)

Pag. S29

Figure S26. ^{13}C{^{1}H} DEPTQ NMR spectrum of [Ru(η^{1}-OAc)(NNN-tpy)((*R*,*R*)-Skewphos)]OAc (**6**)

Pag. S30

Figure S27. ^{1}H-^{1}H COSY 2D NMR spectrum of [Ru(η^{1}-OAc)(NNN-tpy)((*R*,*R*)-Skewphos)]OAc (**6**)

Pag. S31

Figure S28. ^{1}H-^{13}C HSQC 2D NMR spectrum of [Ru(η^1-OAc)(NNN-tpy)((*R*,*R*)-Skewphos)]OAc (**6**) Pag. S32

Figure S29. ^{1}H-^{13}C HMBC 2D NMR spectrum of [Ru(η^1-OAc)(NNN-tpy)((*R*,*R*)-Skewphos)]OAc (**6**) Pag. S33

Figure S30. ^{1}H-^{31}P HMBC 2D NMR spectrum of [Ru(η^1-OAc)(NNN-tpy)((*R*,*R*)-Skewphos)]OAc (**6**) Pag. S34

Figure S31. ^{1}H-^{1}H NOESY 2D NMR spectrum of [Ru(η^1-OAc)(NNN-tpy)((*R*,*R*)-Skewphos)]OAc (**6**) Pag. S35

Figure S32. Evidence of the formation of the [Ru($OAc)_2$((*S*,*S*)-Skewphos)(PPh_3)] intermediate in the reaction of (*S*,*S*)-Skewphos with [Ru(η^2-$OAc)_2(PPh_3)_2$] in methanol at reflux in the $^{31}P\{^{1}H\}$ NMR spectrum Pag. S36

Figure S33. Conversion of complex [Ru(η^2-OAc)(NC-tpy)((*S*,*S*)-Skewphos)] (**5**) to the cationic specie [Ru(η^1-OAc)(NNN-tpy)((*S*,*S*)-Skewphos)]OAc (**7**) promoted by light with acetic acid in the $^{31}P\{^{1}H\}$ NMR spectra in CD_3OD Pag. S37

Figure S34. GC-FID chromatogram of the reaction mixture of the enantioselective photocatalytic TH of acetophenone promoted by complex **7** at S/C 1000 Pag. S38

Figure S35. Comparison between the GC-FID chromatograms of the enantioselective photocatalytic TH of acetophenone promoted by complex **6** (A) and **7** (B) at S/C 1000 Pag. S39

Figure S36. $^{31}P\{^{1}H\}$ NMR spectrum of [Ru(O*i*Pr)(NNN-tpy)((*S*,*S*)-Skewphos)](O*i*Pr) (**A**) in 2-propanol-d^8 obtained after visible light irradiation from complex **7** Pag. S40

Figure S37. ^{1}H NMR spectrum with evidences of the formation of [RuH(NNN-tpy)((*S*,*S*)-Skewphos)](O*i*Pr) (**B**) in 2-propanol/toluene-d^8 Pag. S41

Figure S38. Effect of complexes **1** and **4** on ATC cell viability in SW1736, 8505C and Nthy-ori 3-1 cells Pag. S42

Figure S39. Effect of complexes **5** and **6** on ATC cell viability in SW1736, 8505C and Nthy-ori 3-1

cells Pag. S43

Figure S40. Effect of complexes **7** and [RuCl(NNN-tpy)((*R*,*R*)-Skewphos)]Cl on ATC cell viability in SW1736, 8505C and Nthy-ori 3-1 cells Pag. S44

Figure S41. Effect of complex [RuCl(NNN-tpy)((*S*,*S*)-Skewphos)]PF_6 on ATC cell viability in SW1736, 8505C and Nthy-ori 3-1 cells Pag. S45

Table S1. Statistical analysis for tested complexes Pag. S46

Single crystal X-ray structure determination of Compound 1. General data. Pag. S47

Figure S42. Molecular structure of complex **1** Pag. S48

Single crystal X-ray structure determination of complex 1. Detailed crystallographic data. Pag. S49

References Pag. S51

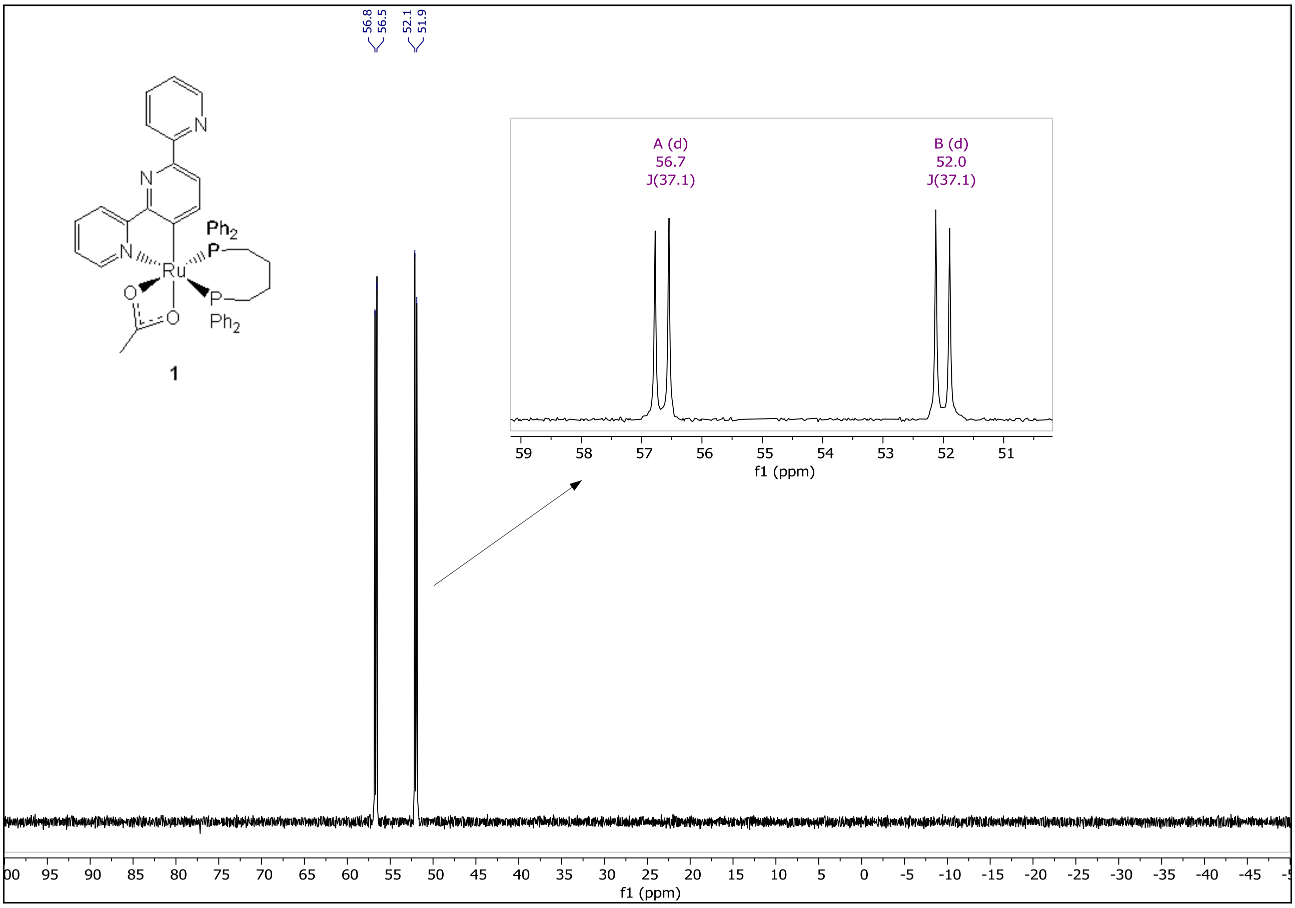

Figure S1. $^{31}P\{^{1}H\}$ NMR spectrum (162.0 MHz) of [Ru(η^2-OAc)(NC-tpy)(dppb)] (**1**) in CD_2Cl_2 at 25 °C.

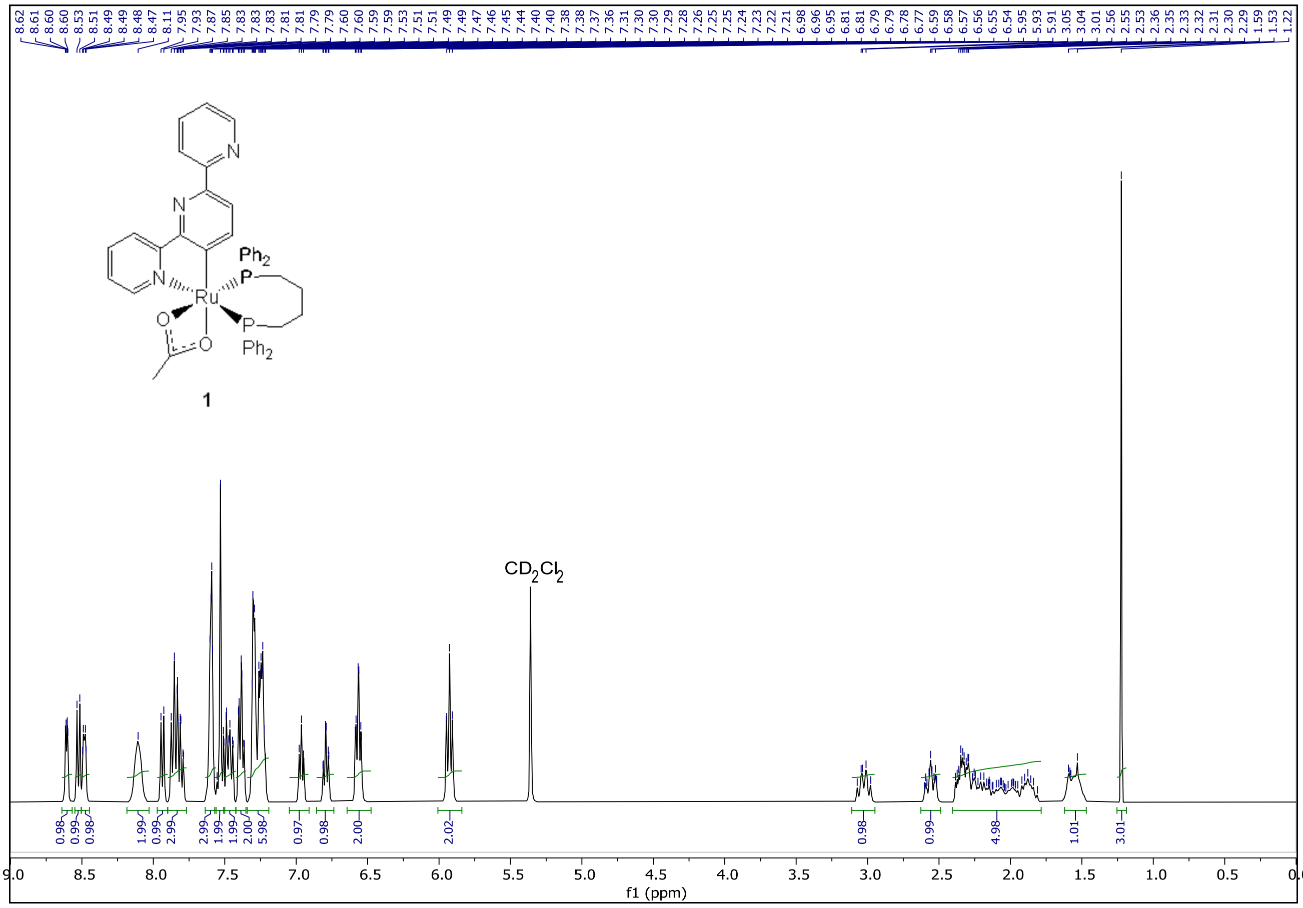

Figure S2. ^{1}H NMR spectrum (400.1 MHz) of [Ru(η^2-OAc)(NC-tpy)(dppb)] (**1**) in CD_2Cl_2 at 25 °C.

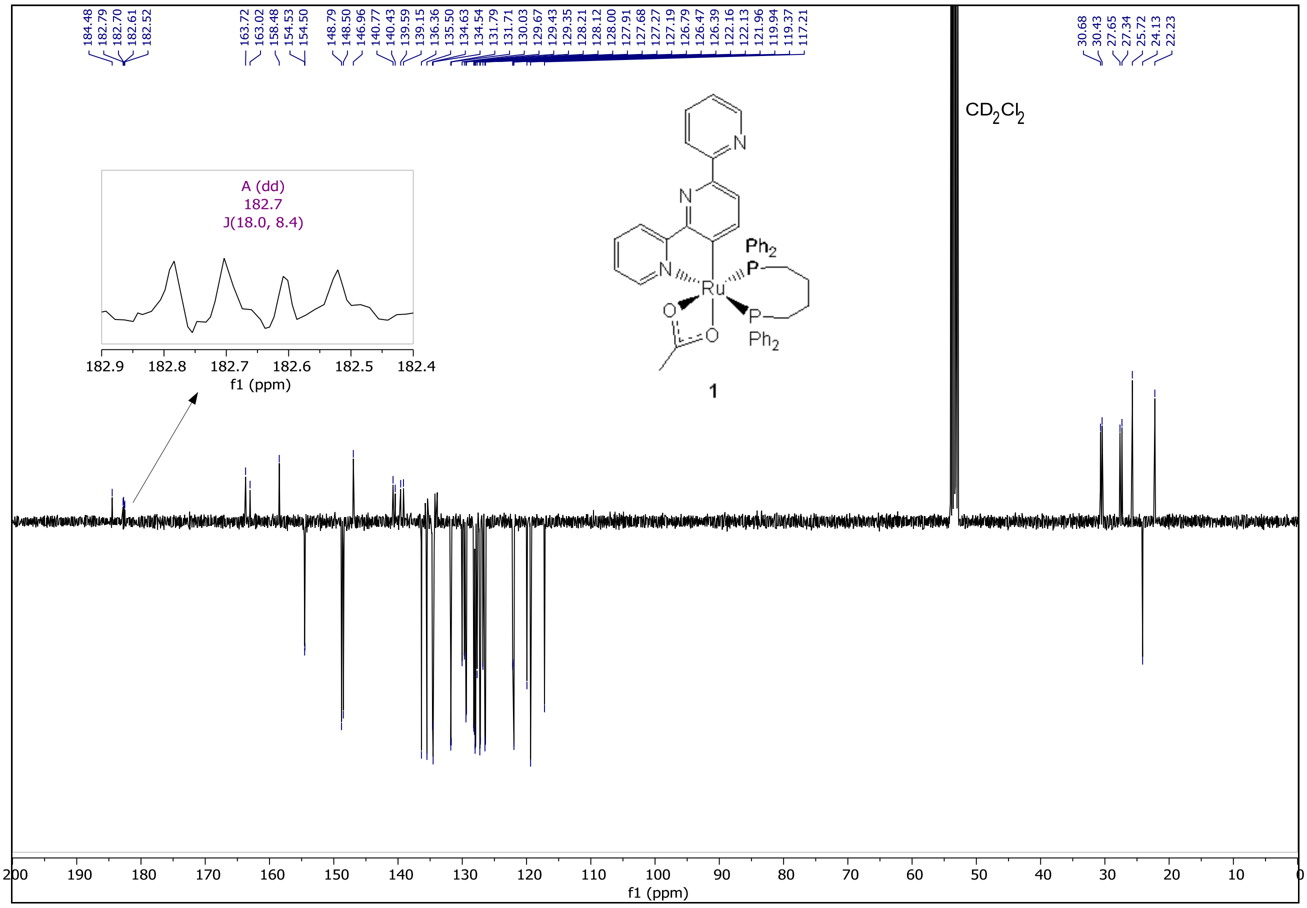

Figure S3. $^{13}C\{^{1}H\}$ DEPTQ NMR spectrum (100.6 MHz) of [Ru(η^2-OAc)(NC-tpy)(dppb)] (**1**) in CD_2Cl_2 at 25 °C.

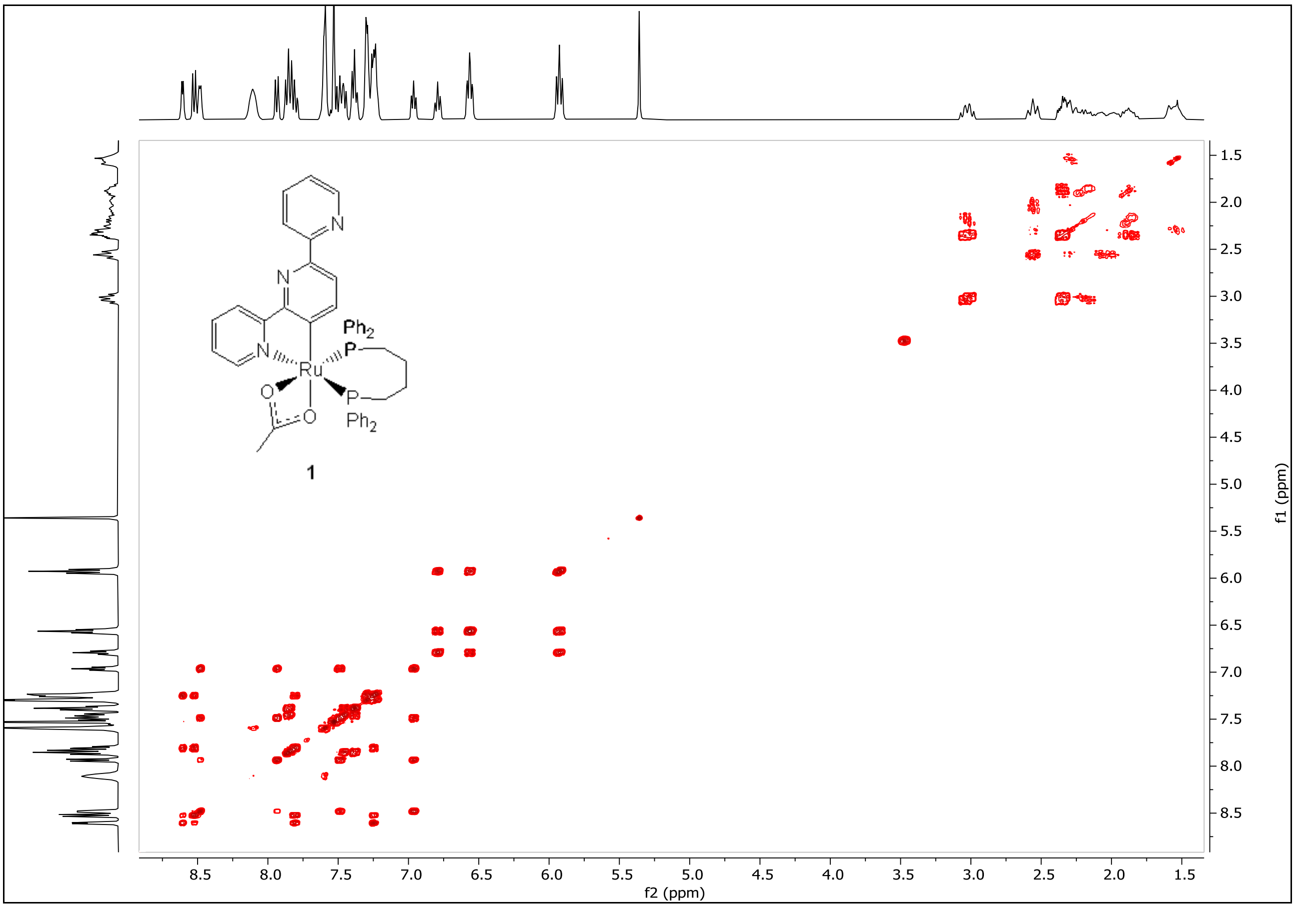

Figure S4. ^{1}H-^{1}H COSY 2D NMR spectrum of [Ru(η^2-OAc)(NC-tpy)(dppb)] (**1**) in CD_2Cl_2 at 25 °C.

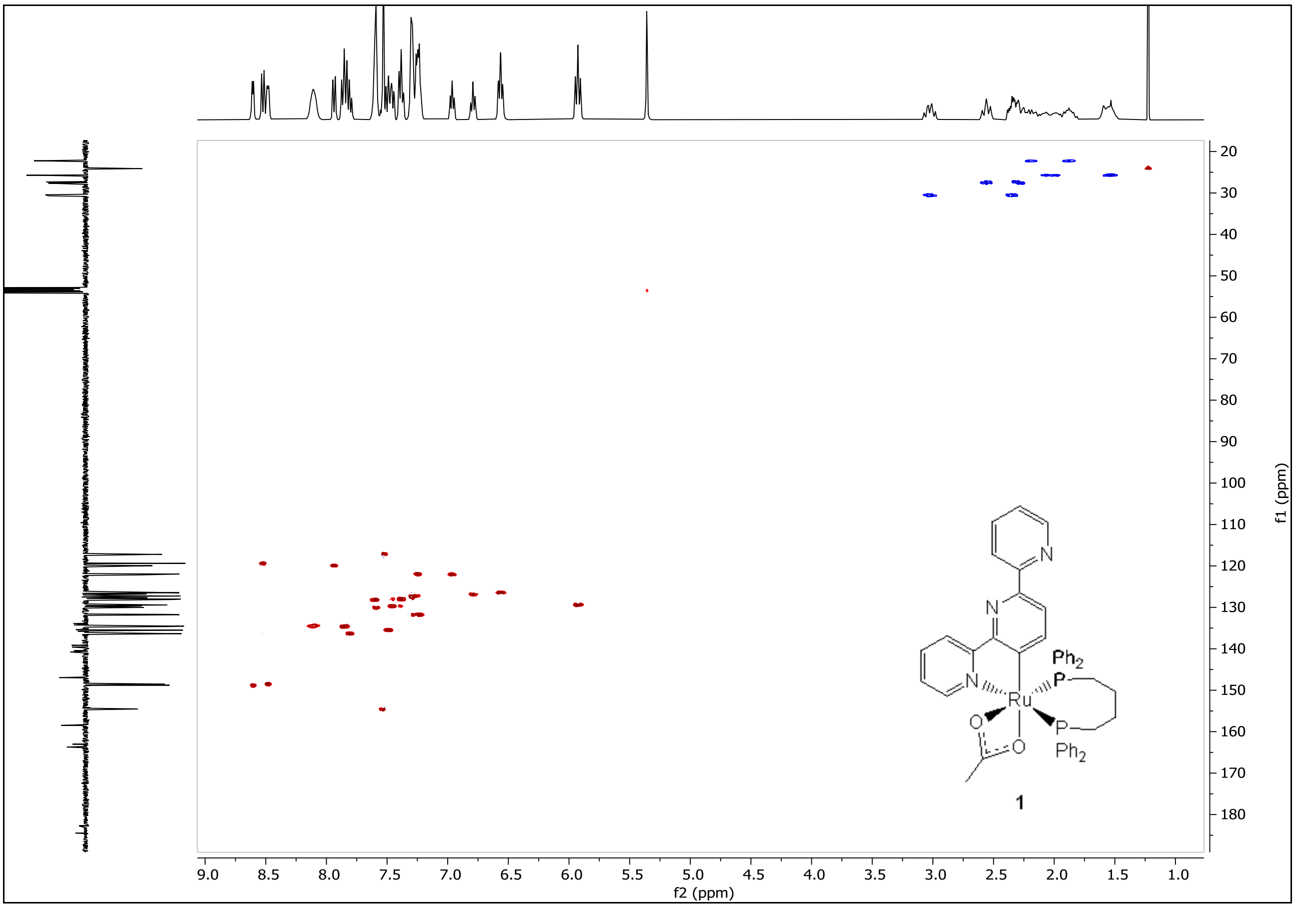

Figure S5. ^{1}H-^{13}C HSQC 2D NMR spectrum of [Ru(η^2-OAc)(NC-tpy)(dppb)] (**1**) in CD_2Cl_2 at 25 °C.

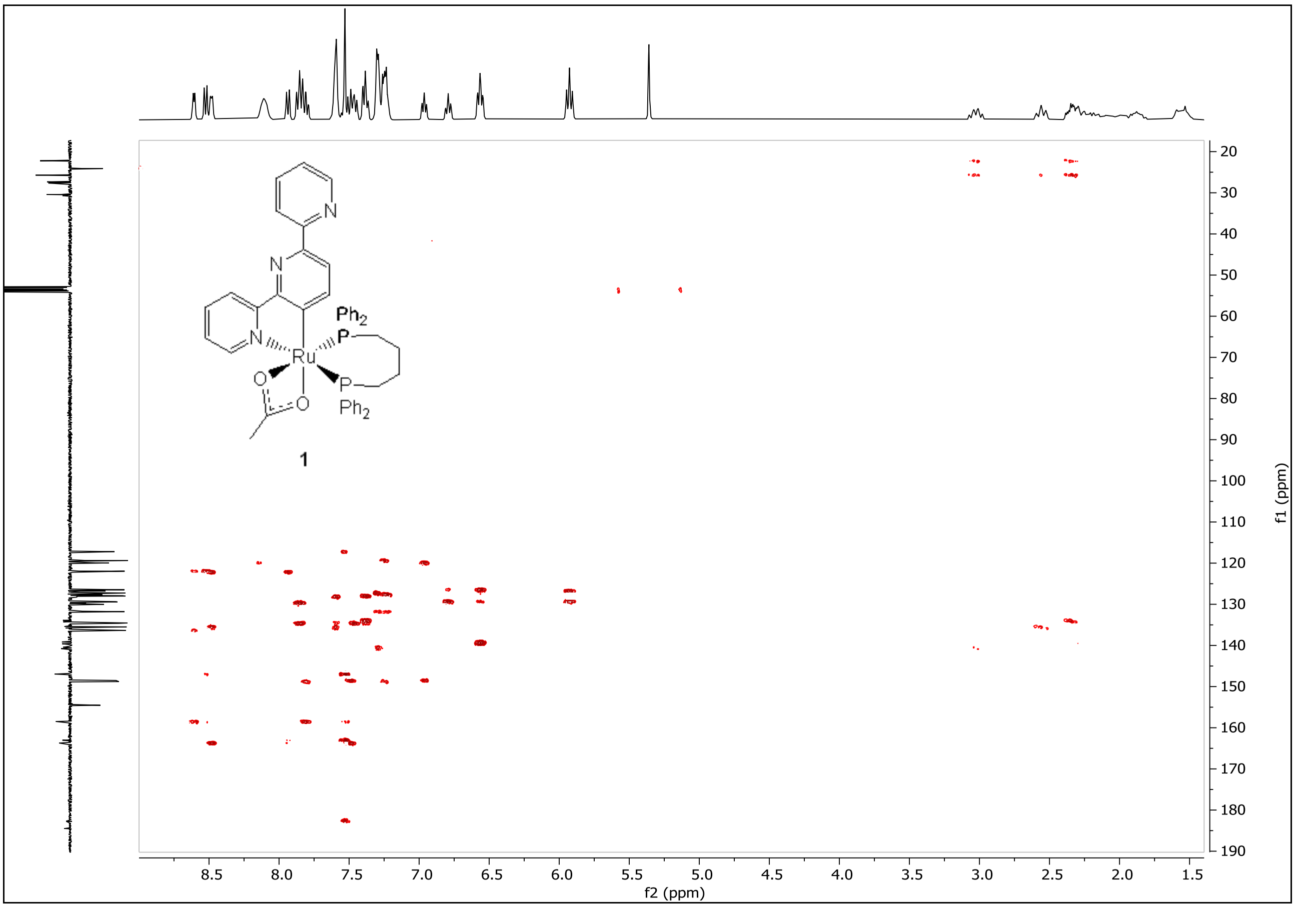

Figure S6. ^{1}H-^{13}C HMBC 2D NMR spectrum of [Ru(η^2-OAc)(NC-tpy)(dppb)] (**1**) in CD_2Cl_2 at 25 °C.

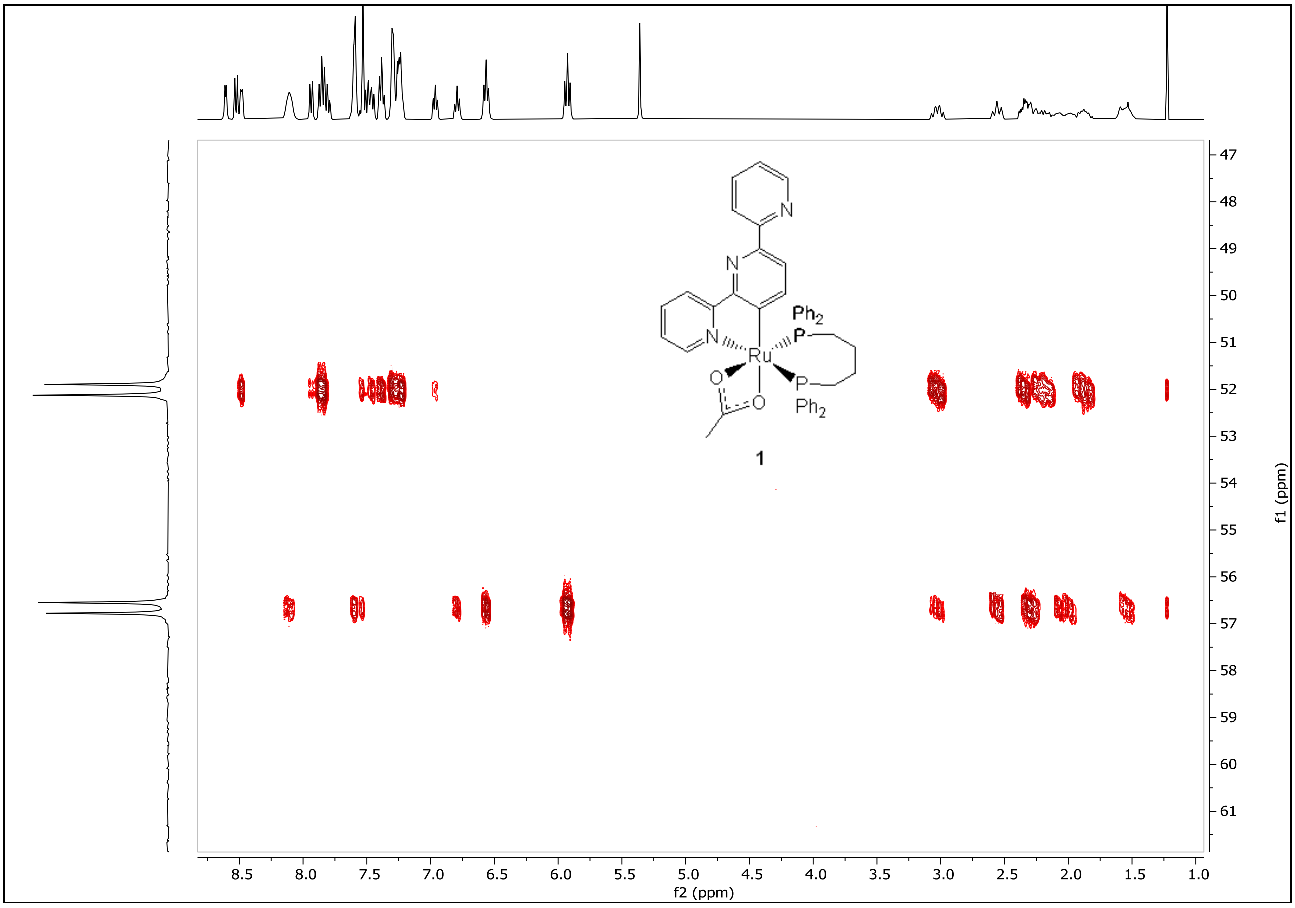

Figure S7. ^{1}H-^{31}P HMBC 2D NMR spectrum of [Ru(η^2-OAc)(NC-tpy)(dppb)] (**1**) in CD_2Cl_2 at 25 °C.

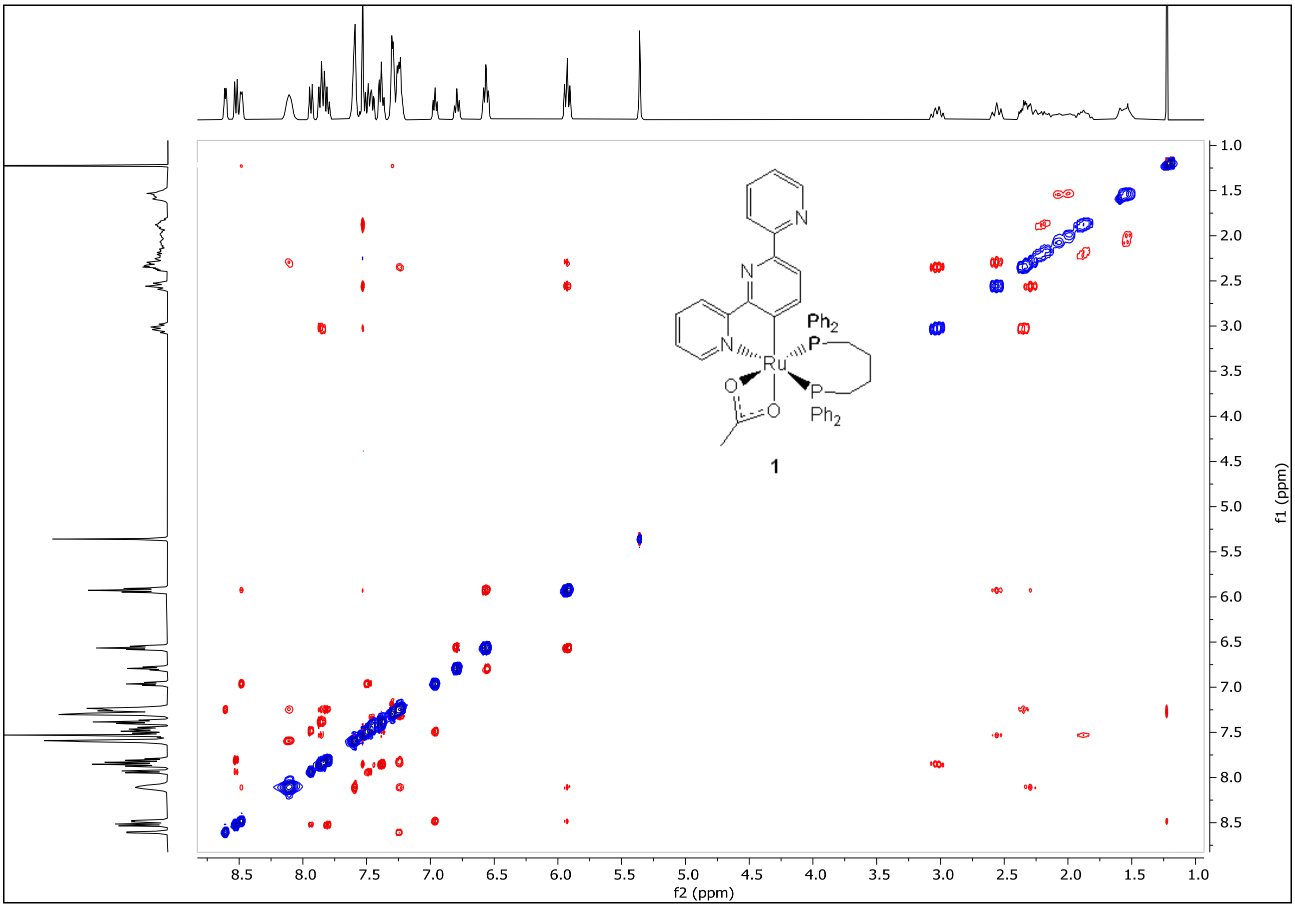

Figure S8. ^{1}H-^{1}H NOESY 2D NMR spectrum of [Ru(η^2-OAc)(NC-tpy)(dppb)] (**1**) in CD_2Cl_2 at 25 °C.

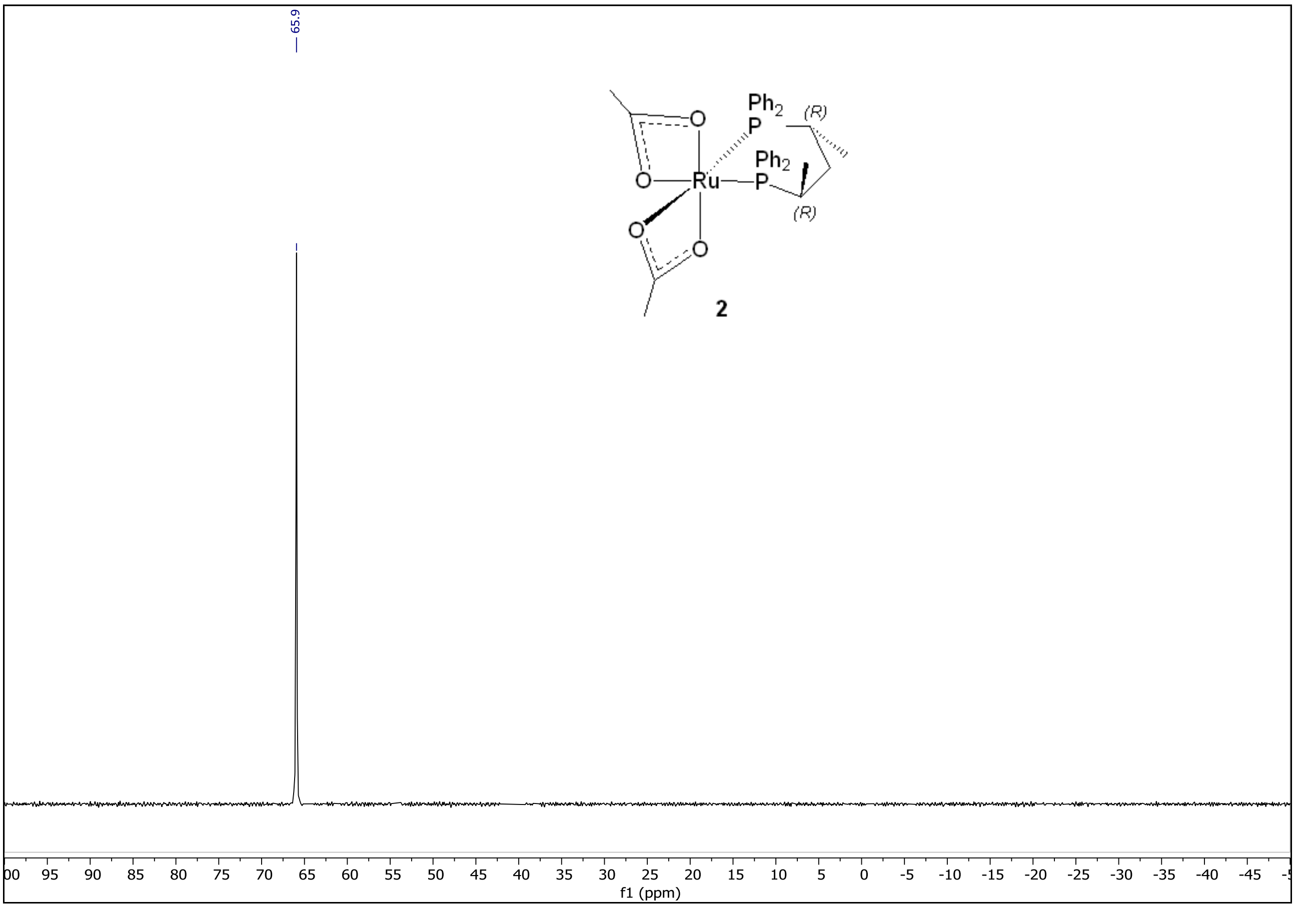

Figure S9. $^{31}P\{^1H\}$ NMR spectrum (162.0 MHz) of [Ru(η^2-OAc)$_2$((*R*,*R*)-Skewphos)] (**2**) in CD_3OD at 25 °C.

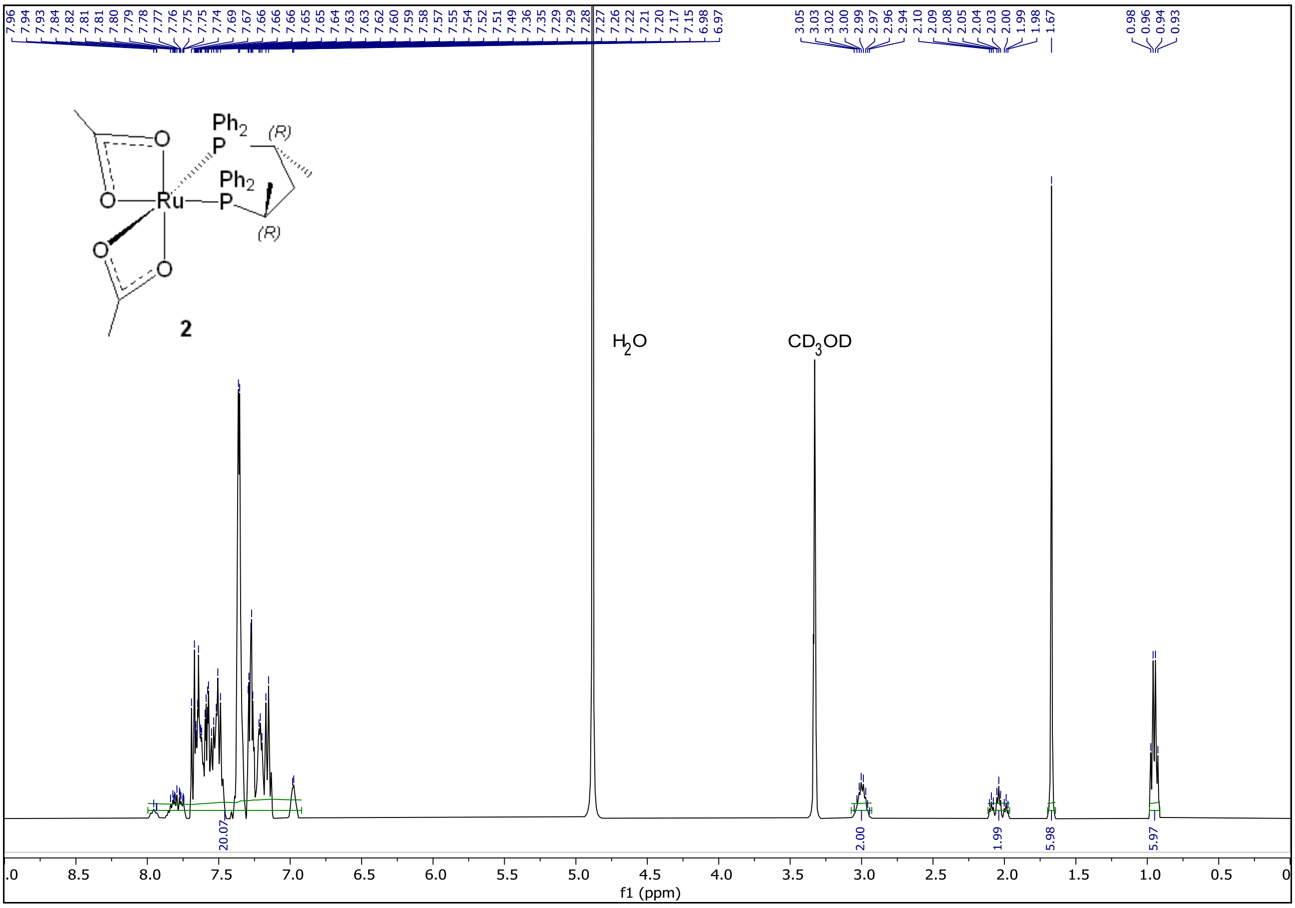

Figure S10. ^{1}H NMR spectrum (400.1 MHz) of [Ru(η^2-OAc)$_2$((*R*,*R*)-Skewphos)] (**2**) in CD_3OD at 25 °C.

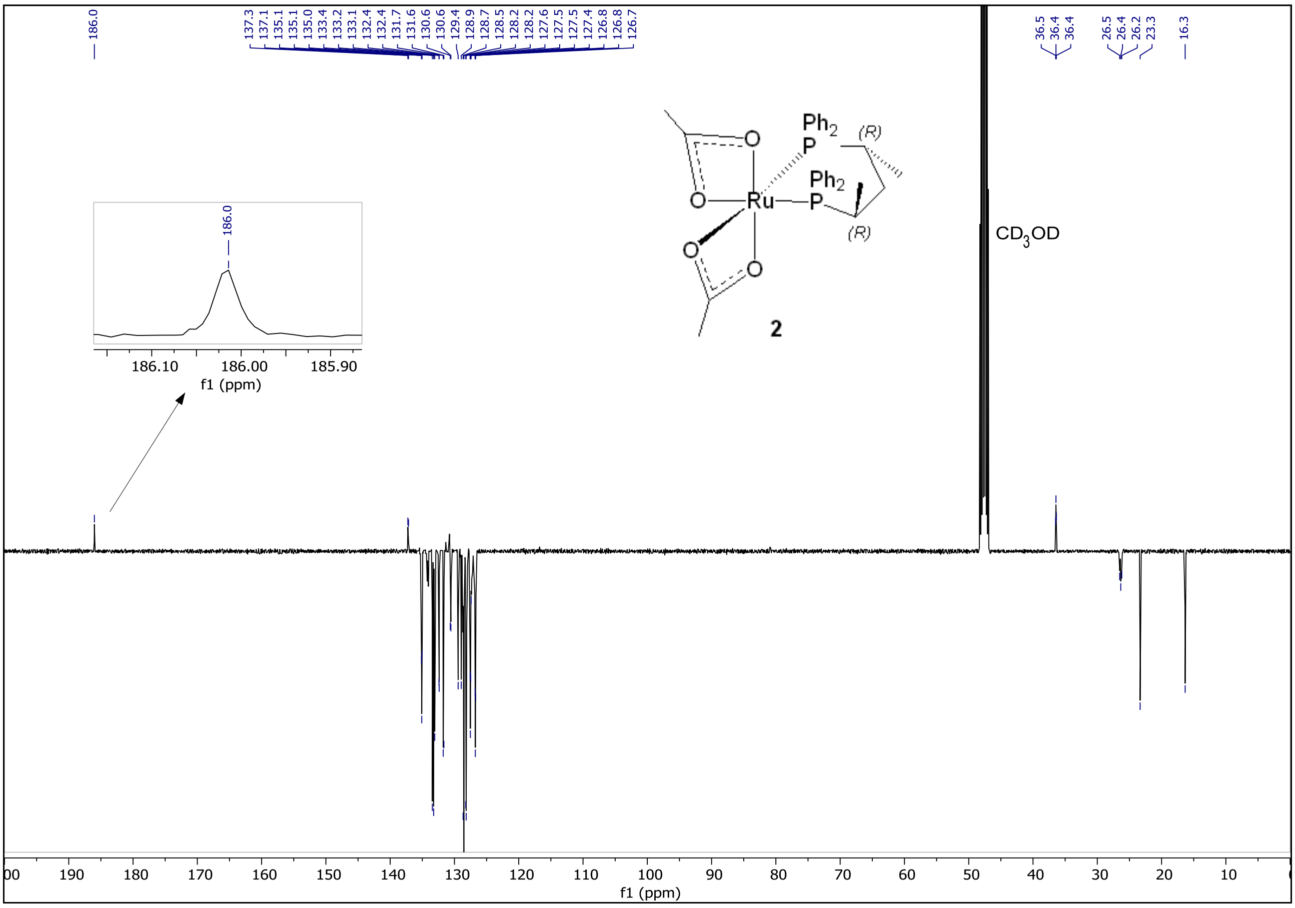

Figure S11. $^{13}C\{^1H\}$ NMR spectrum (100.6 MHz) of [Ru(η^2-OAc)$_2$((*R*,*R*)-Skewphos)] (**2**) in CD_3OD at 25 °C.

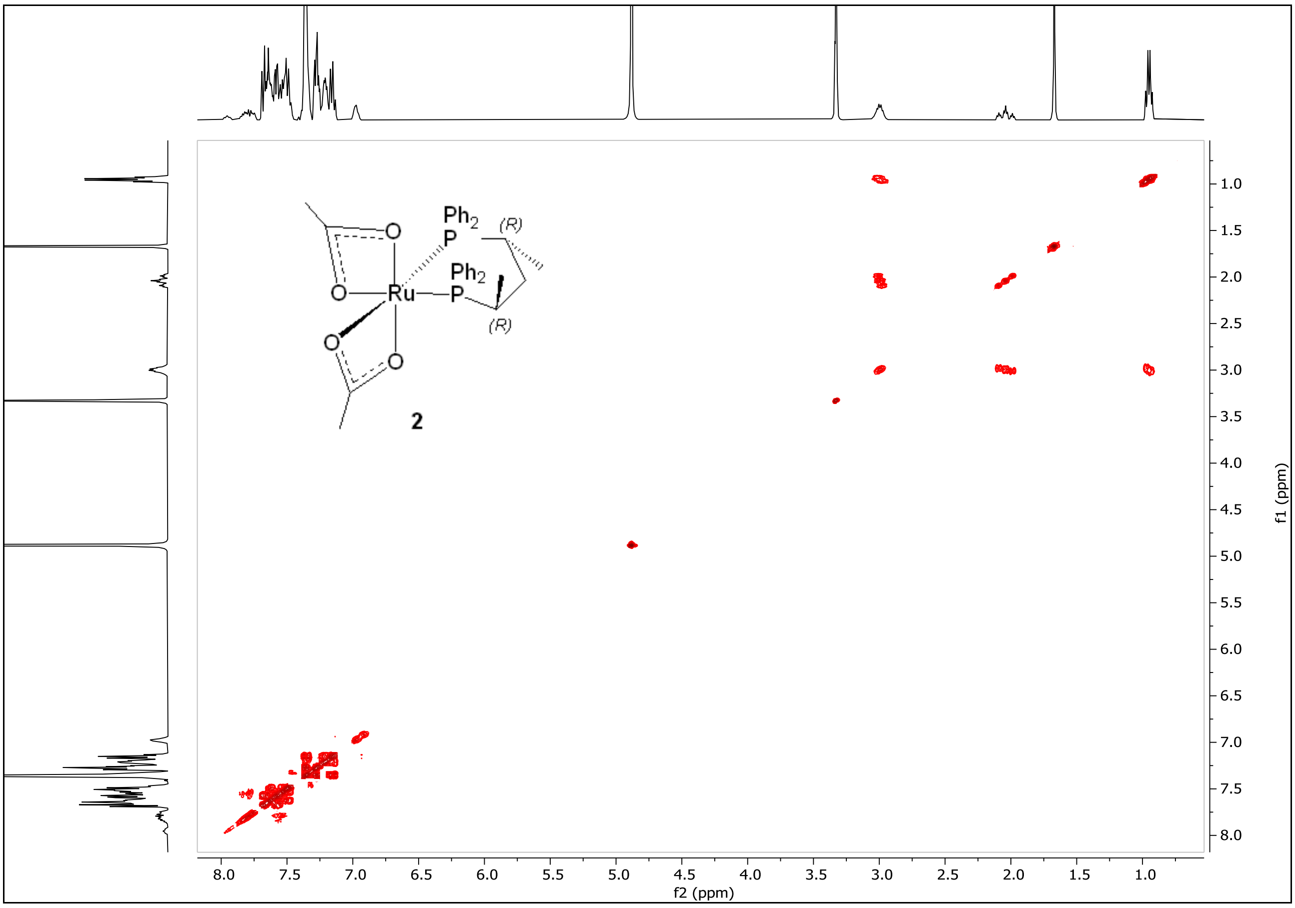

Figure S12. ^{1}H-^{1}H COSY 2D NMR spectrum of [Ru(η^2-OAc)$_2$((*R*,*R*)-Skewphos)] (**2**) in CD_3OD at 25 °C.

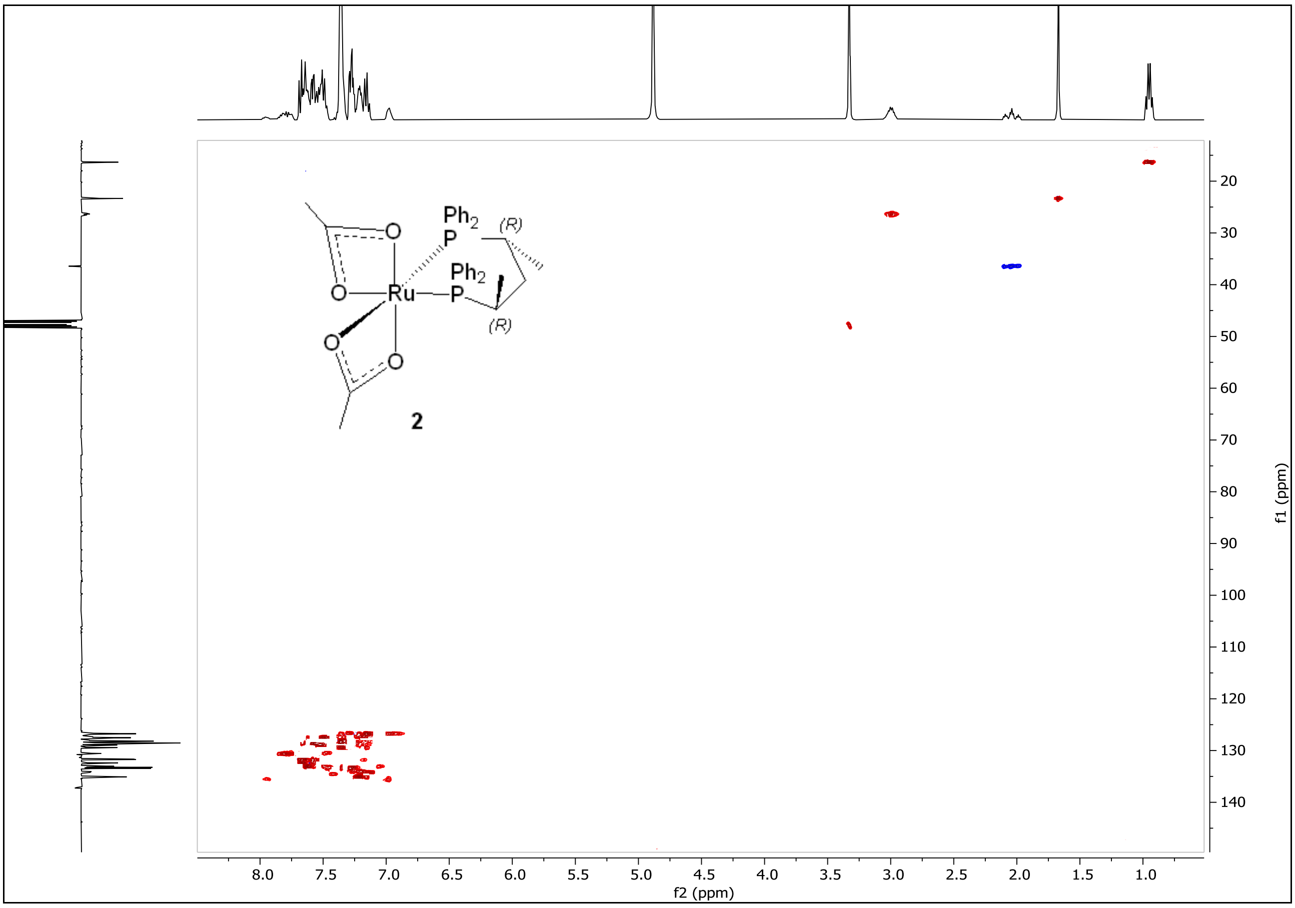

Figure S13. ^{1}H-^{13}C HSQC 2D NMR spectrum of [Ru(η^2-OAc)$_2$((*R*,*R*)-Skewphos)] (**2**) in CD_3OD at 25 °C.

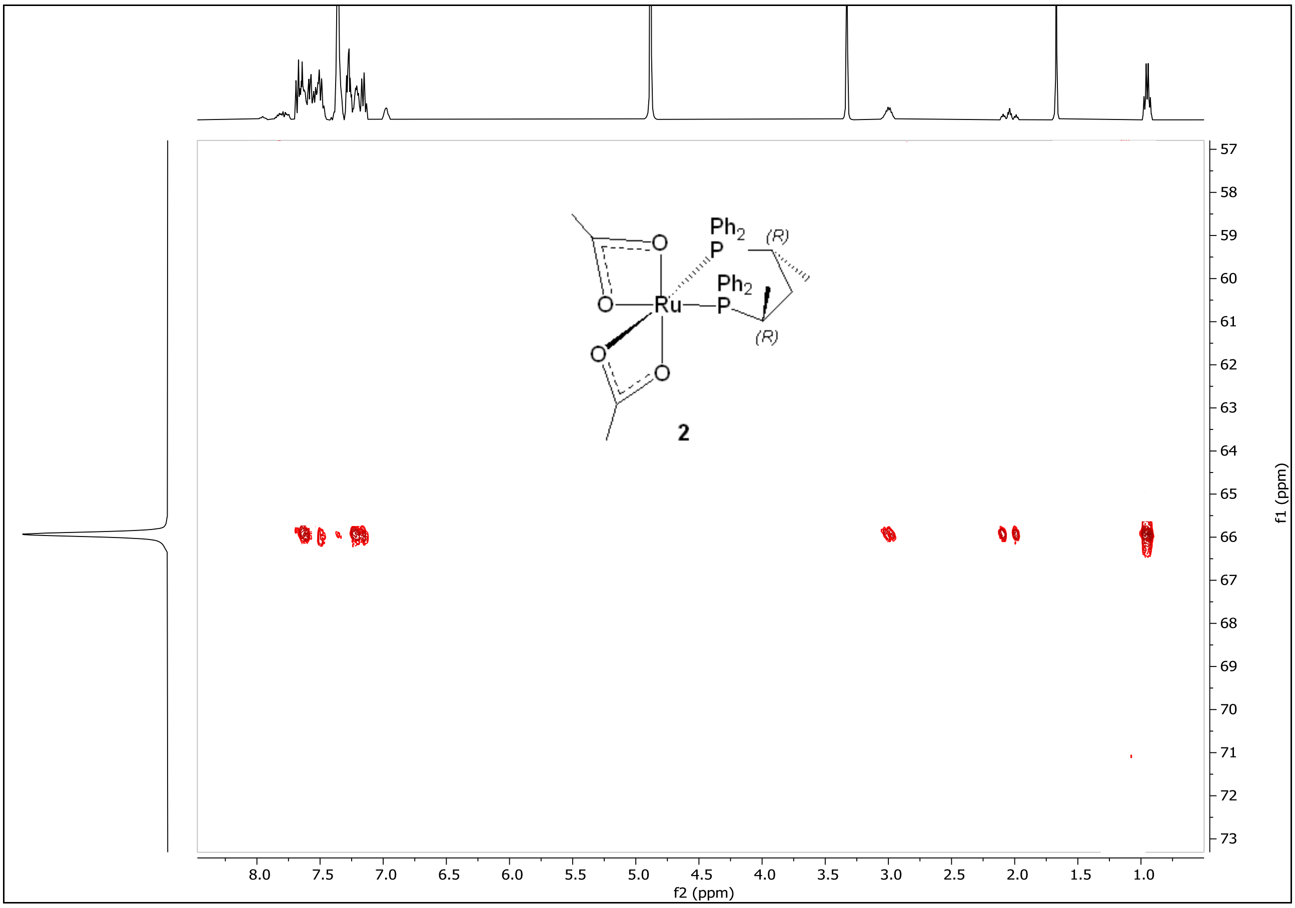

Figure S14. ^1H-^{31}P HMBC 2D NMR spectrum of [Ru(η^2-OAc)$_2$((*R*,*R*)-Skewphos)] (**2**) in CD_3OD at 25 °C.

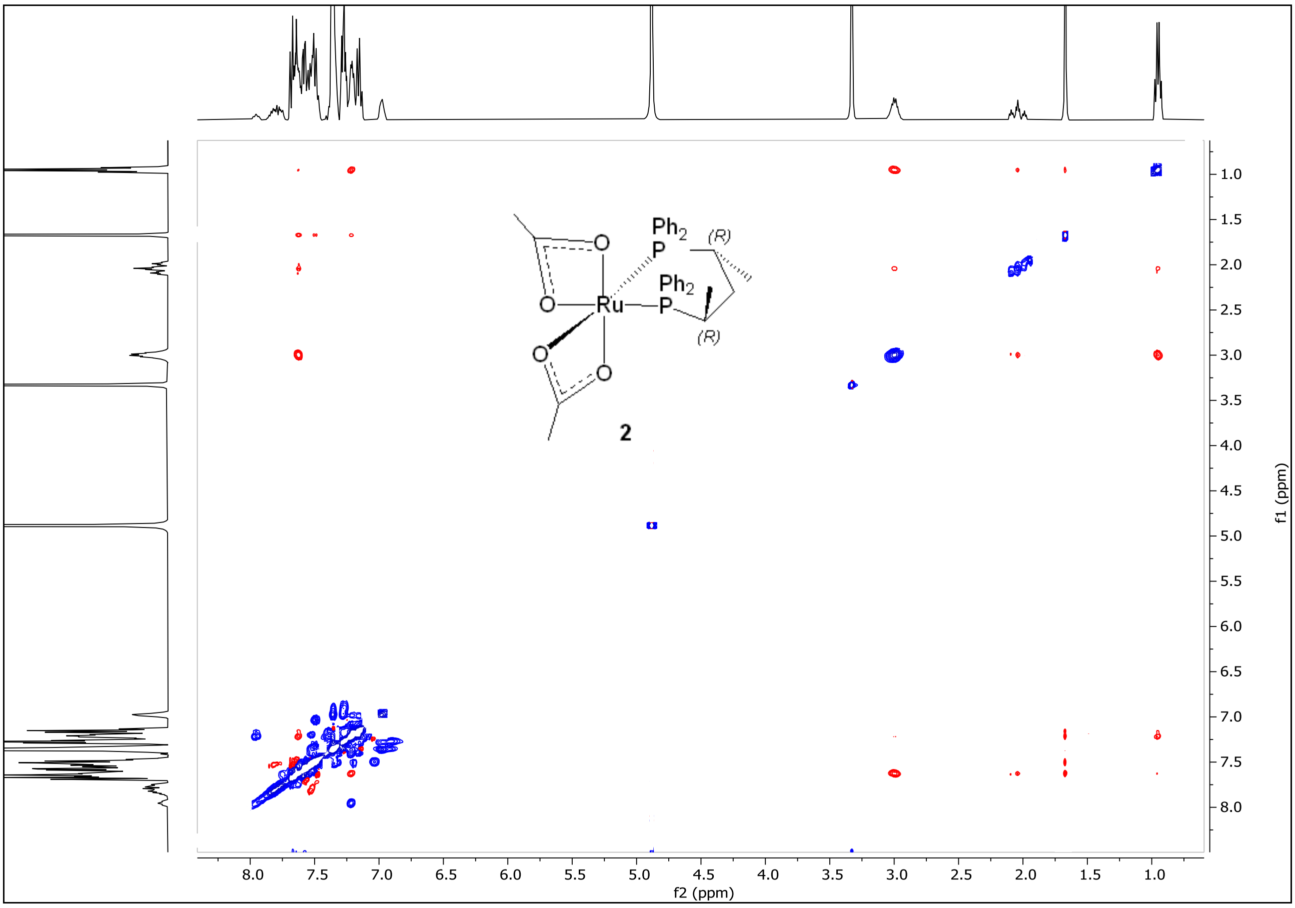

Figure S15. ^1H-^1H NOESY 2D NMR spectrum of [Ru(η^2-OAc)$_2$((*R*,*R*)-Skewphos)] (**2**) in CD_3OD at 25 °C.

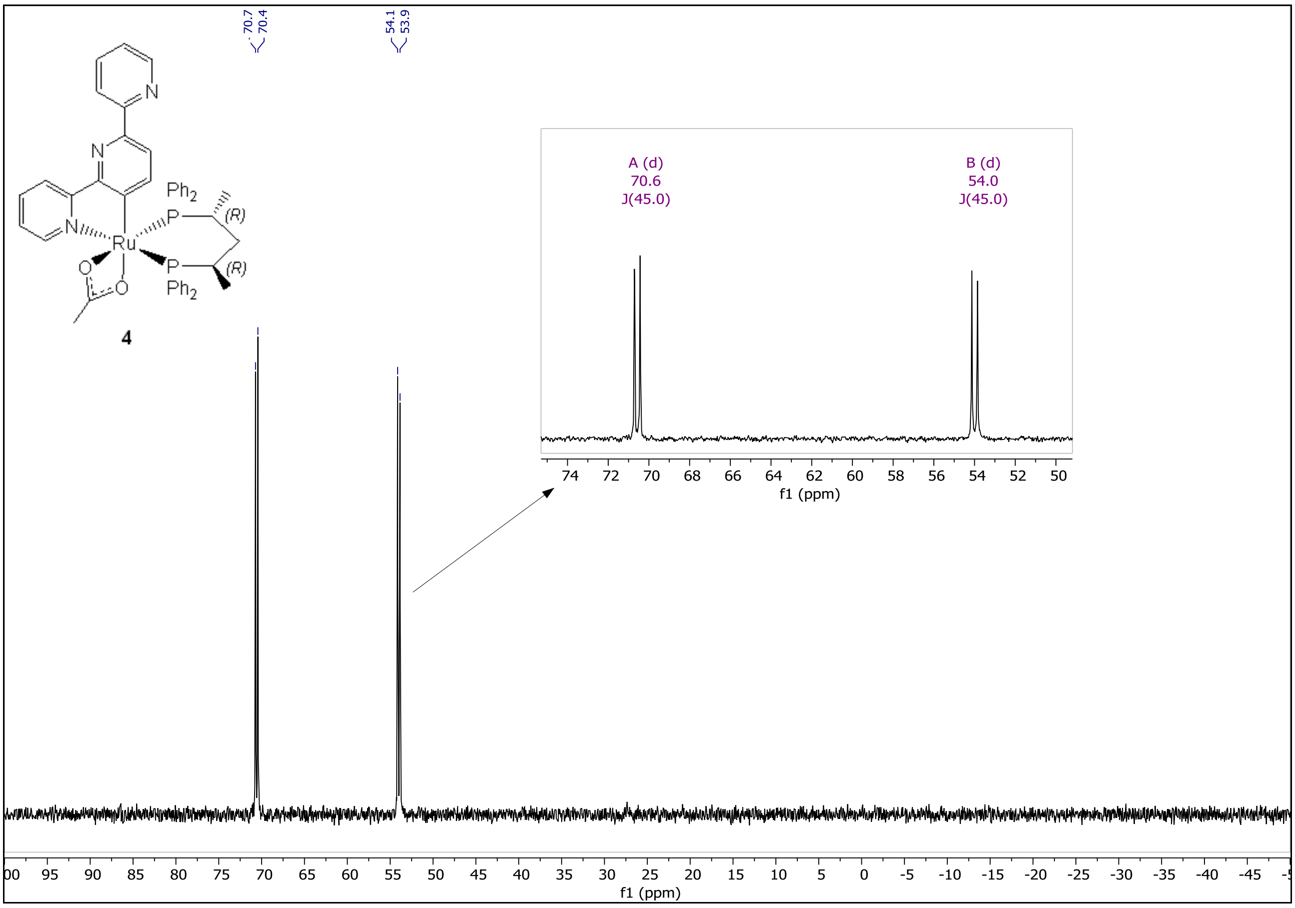

Figure S16. $^{31}P\{^{1}H\}$ NMR spectrum (162.0 MHz) of [Ru(η^2-OAc)(NC-tpy)((*R*,*R*)-Skewphos)] (**4**) in CD_2Cl_2 at 25 °C.

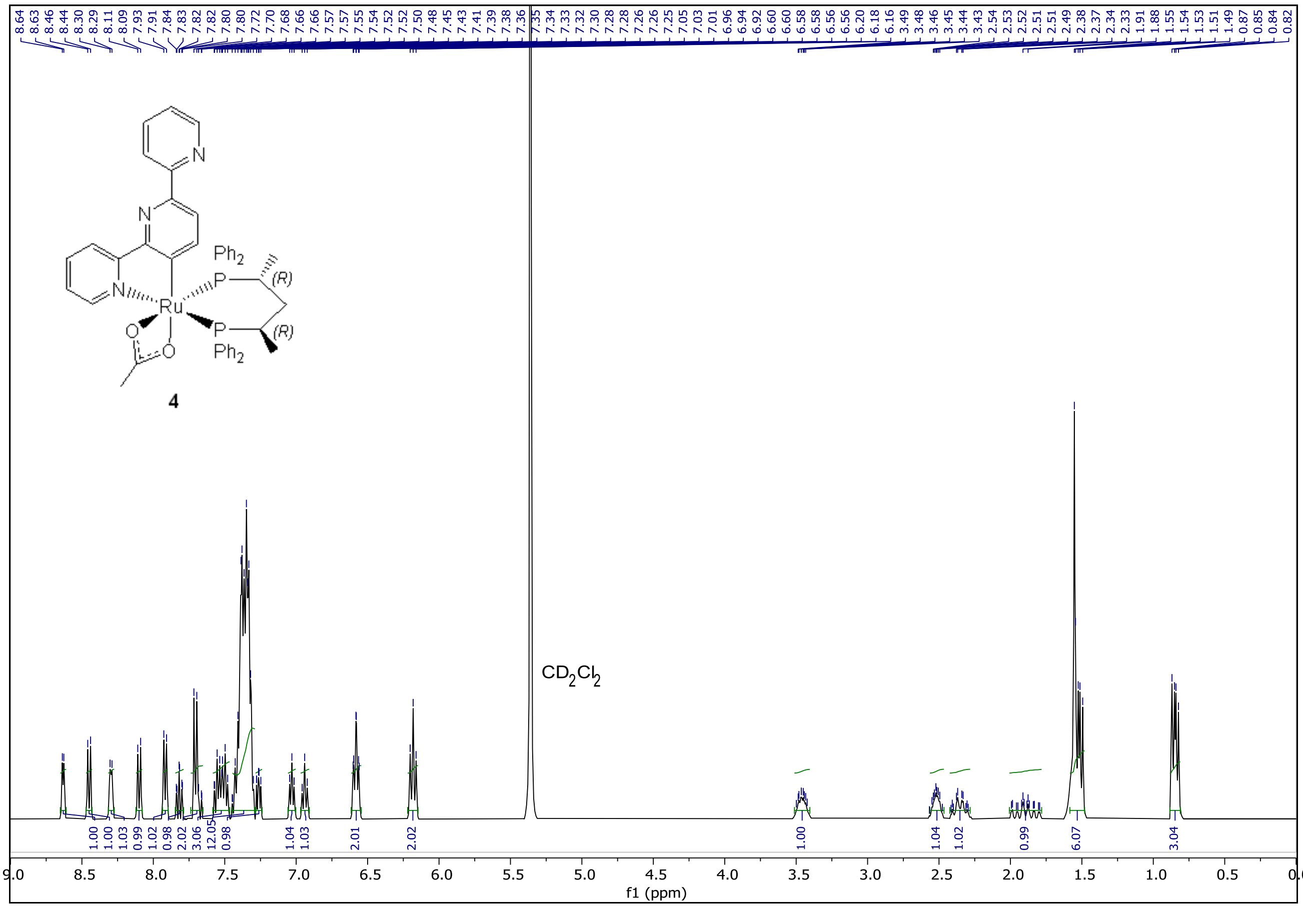

Figure S17. ^{1}H NMR spectrum (400.1 MHz) of [Ru(η^2-OAc)(NC-tpy)((*R*,*R*)-Skewphos)] (**4**) in CD_2Cl_2 at 25 °C.

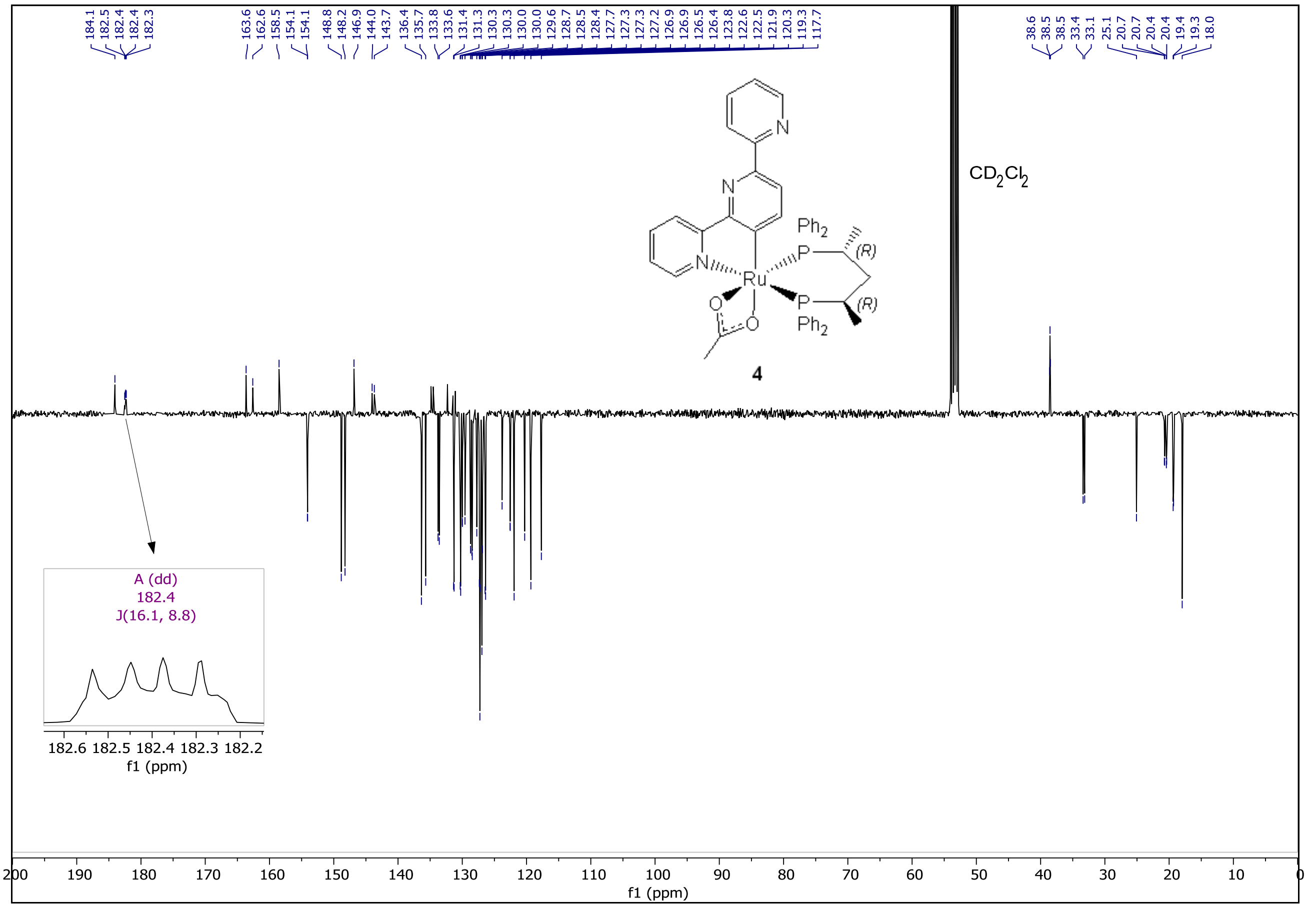

Figure S18. $^{13}C\{^{1}H\}$ DEPTQ NMR spectrum (100.6 MHz) of [Ru(η^2-OAc)(NC-tpy)((*R*,*R*)-Skewphos)] (**4**) in CD_2Cl_2 at 25 °C.

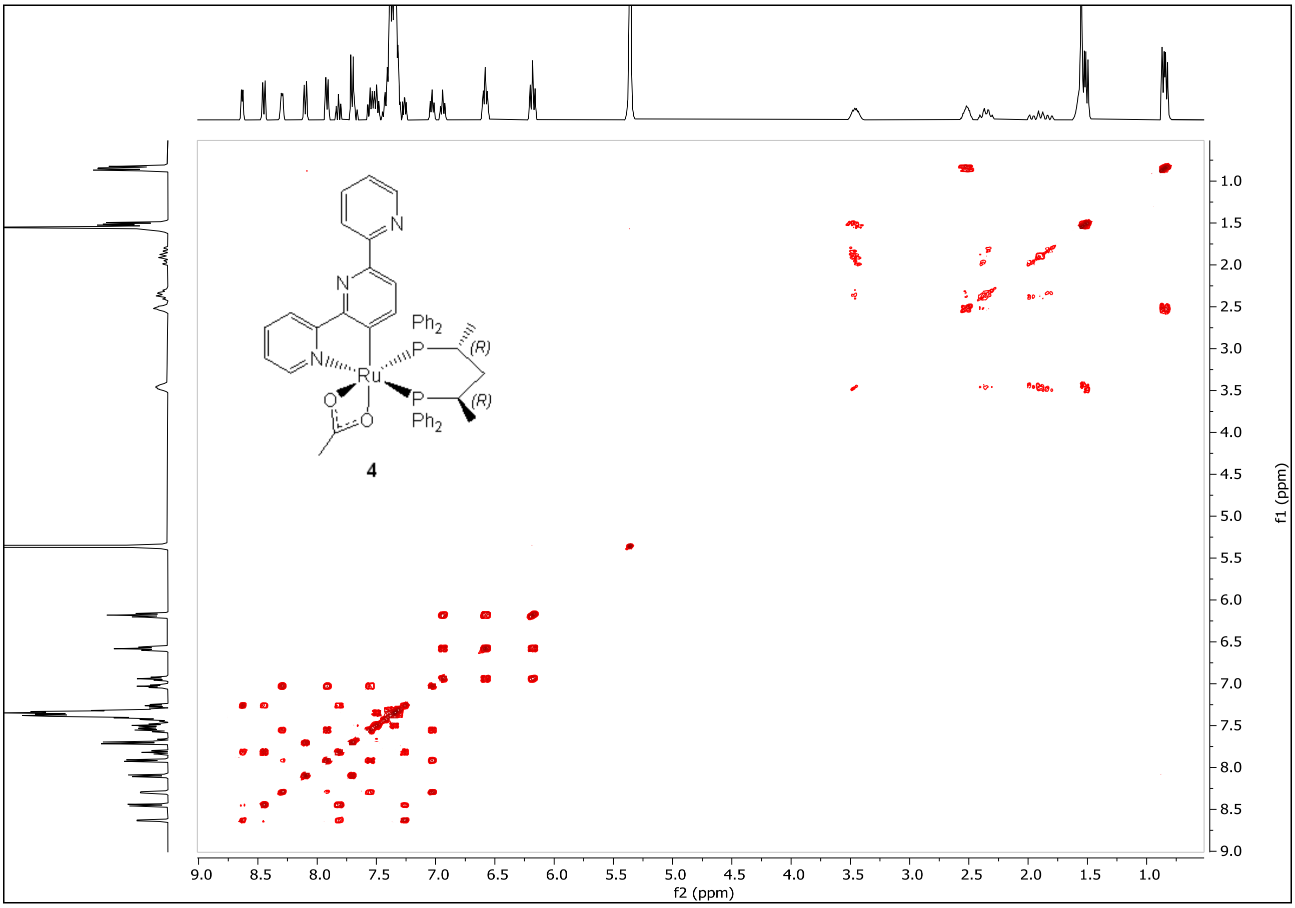

Figure S19. ^{1}H-^{1}H COSY 2D NMR spectrum of [Ru(η^2-OAc)(NC-tpy)((*R*,*R*)-Skewphos)] (**4**) in CD_2Cl_2 at 25 °C.

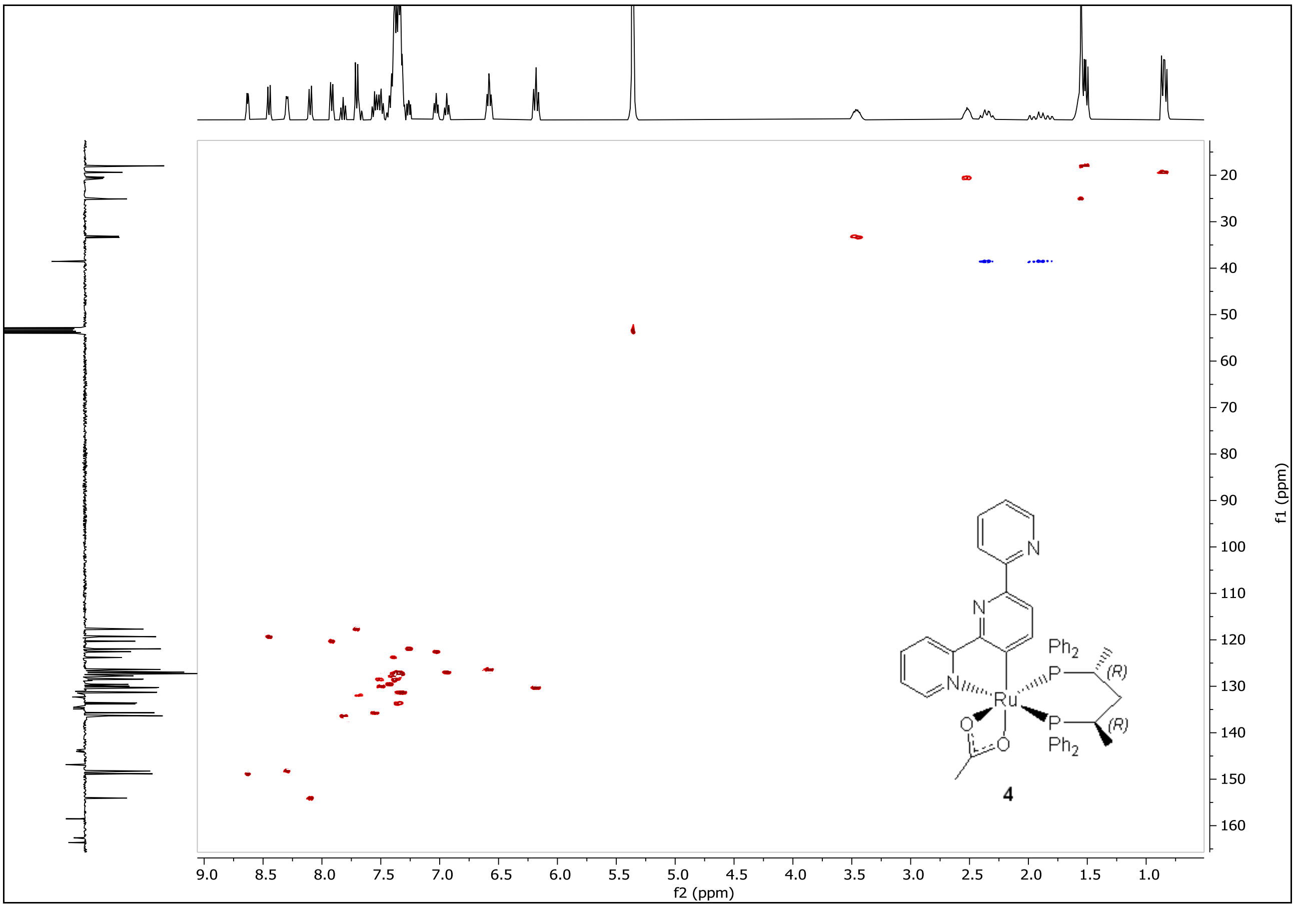

Figure S20. ^{1}H-^{13}C HSQC 2D NMR spectrum of [Ru(η^2-OAc)(NC-tpy)((*R*,*R*)-Skewphos)] (**4**) in CD_2Cl_2 at 25 °C.

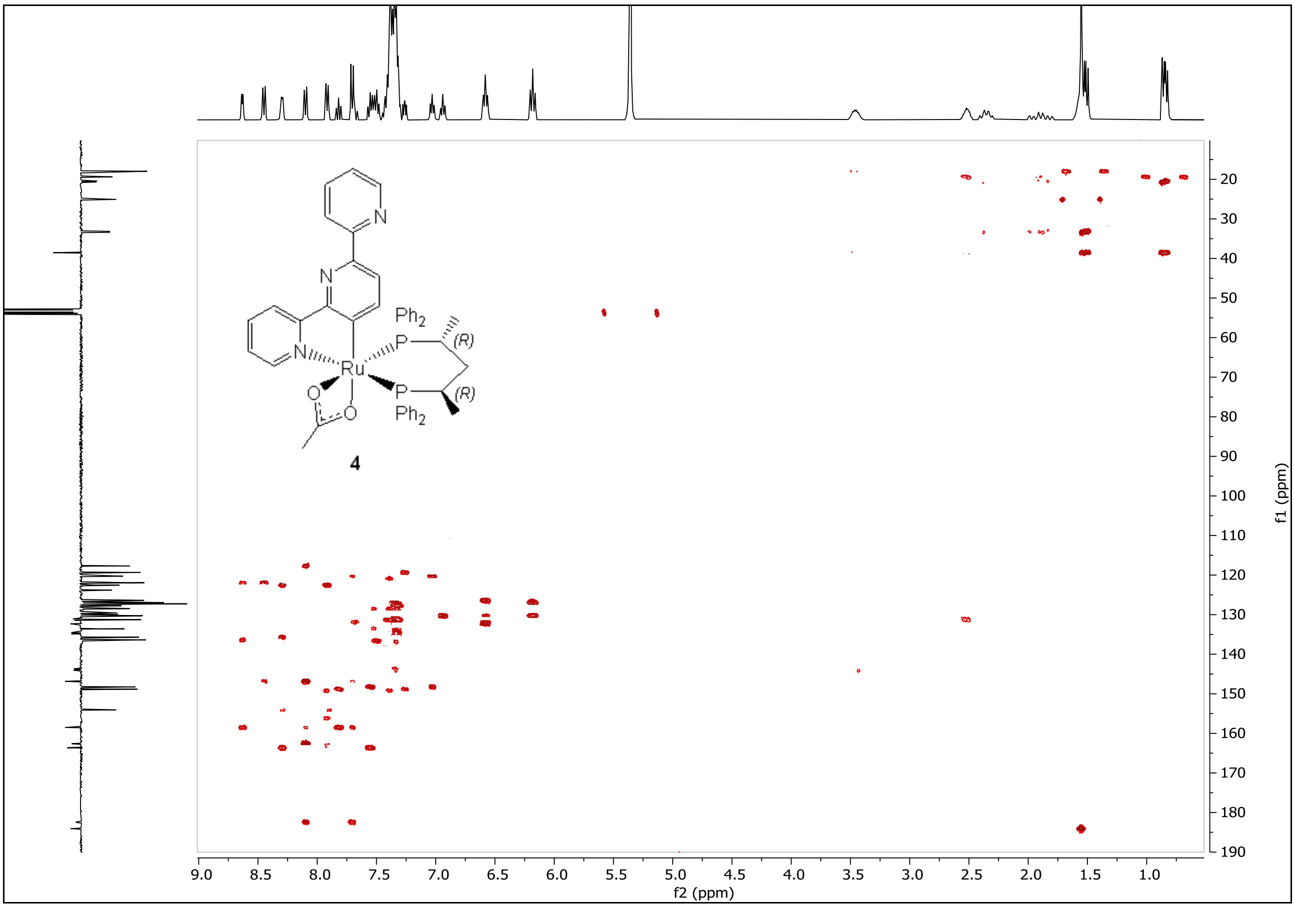

Figure S21. ^{1}H-^{13}C HMBC 2D NMR spectrum of [Ru(η^2-OAc)(NC-tpy)((*R*,*R*)-Skewphos)] (**4**) in CD_2Cl_2 at 25 °C.

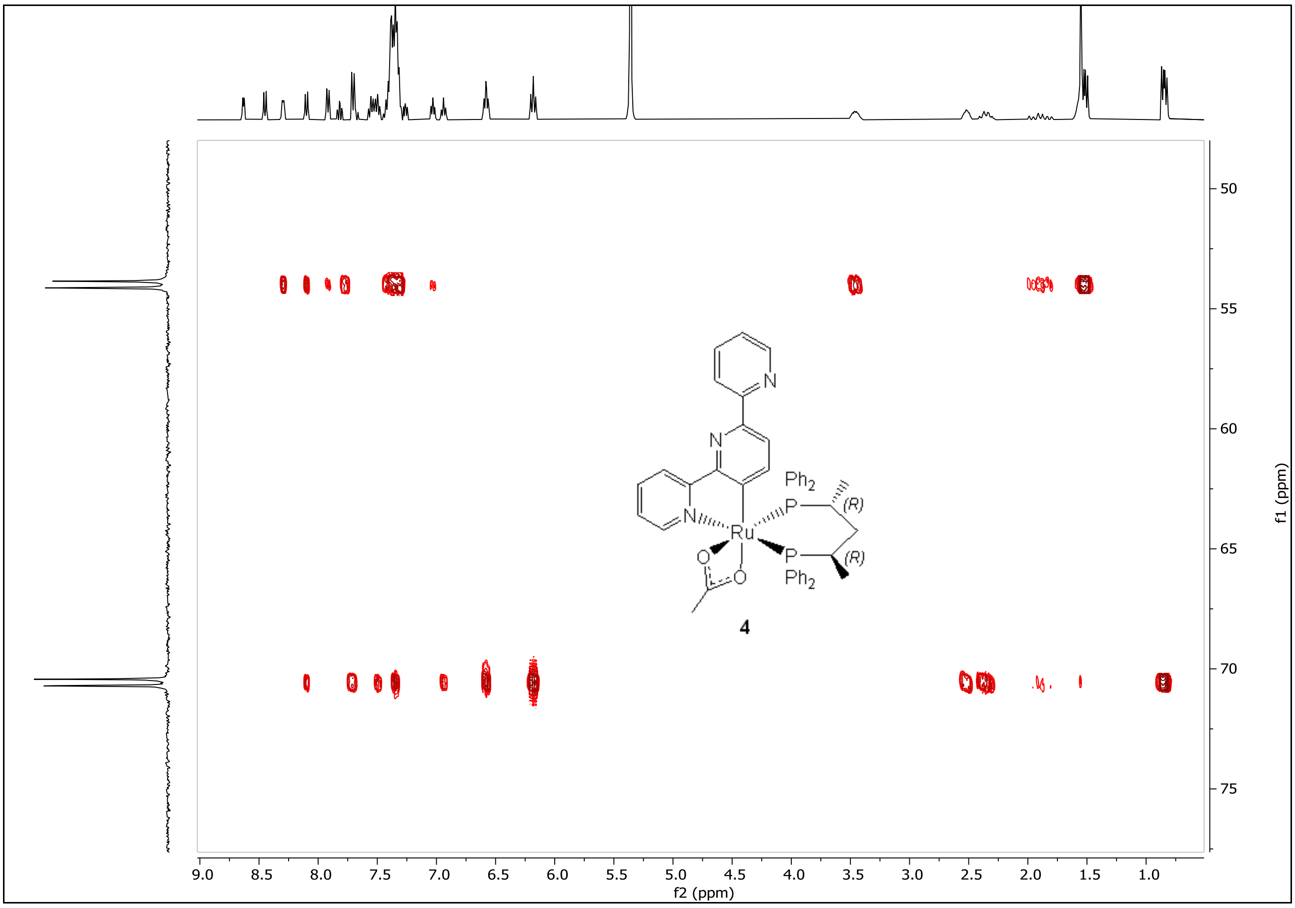

Figure S22. ^{1}H-^{31}P HMBC 2D NMR spectrum of [Ru(η^2-OAc)(NC-tpy)((*R*,*R*)-Skewphos)] (**4**) in CD_2Cl_2 at 25 °C.

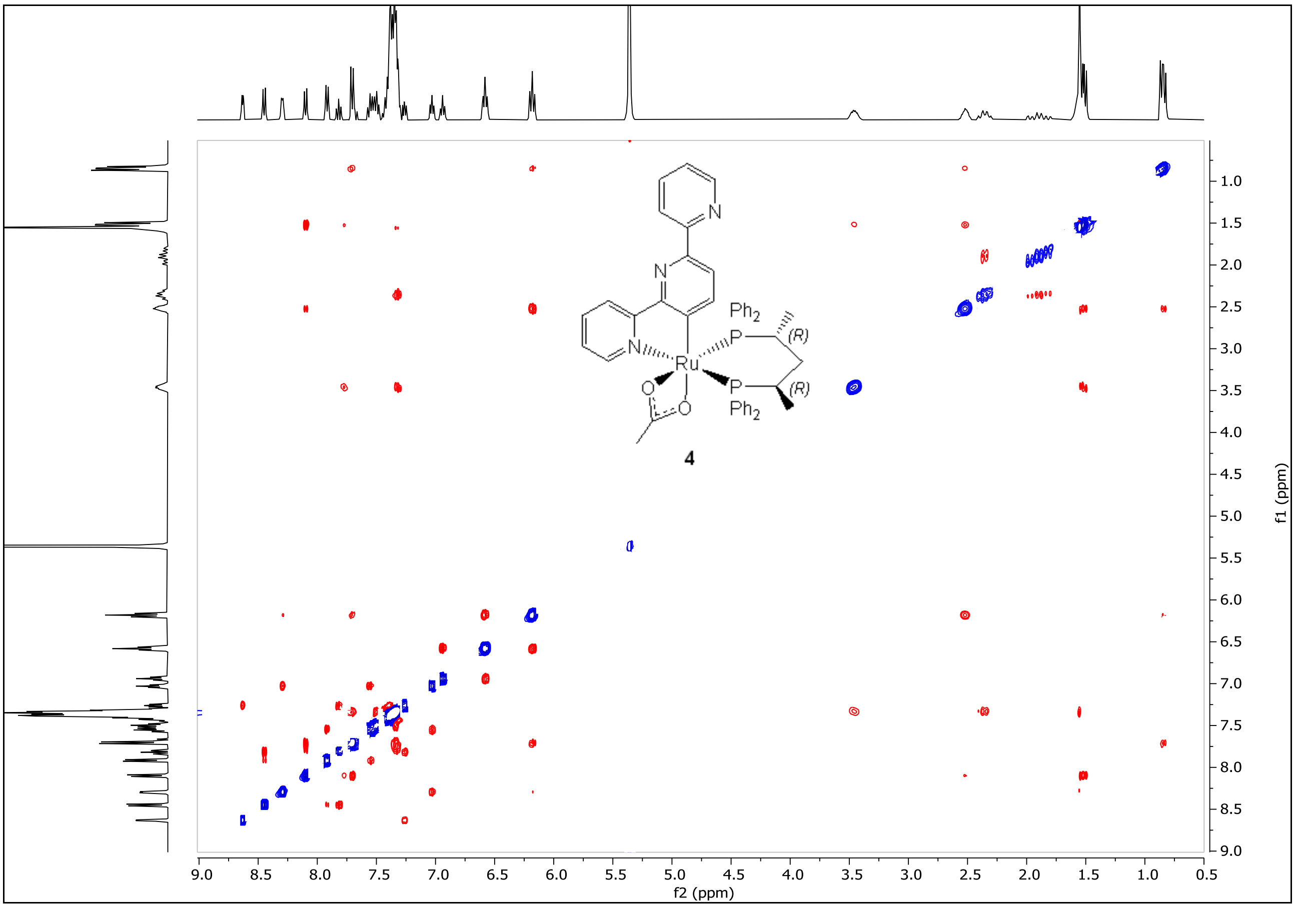

Figure S23. ^{1}H-^{1}H NOESY 2D NMR spectrum of [Ru(η^2-OAc)(NC-tpy)((*R*,*R*)-Skewphos)] (**4**) in CD_2Cl_2 at 25 °C.

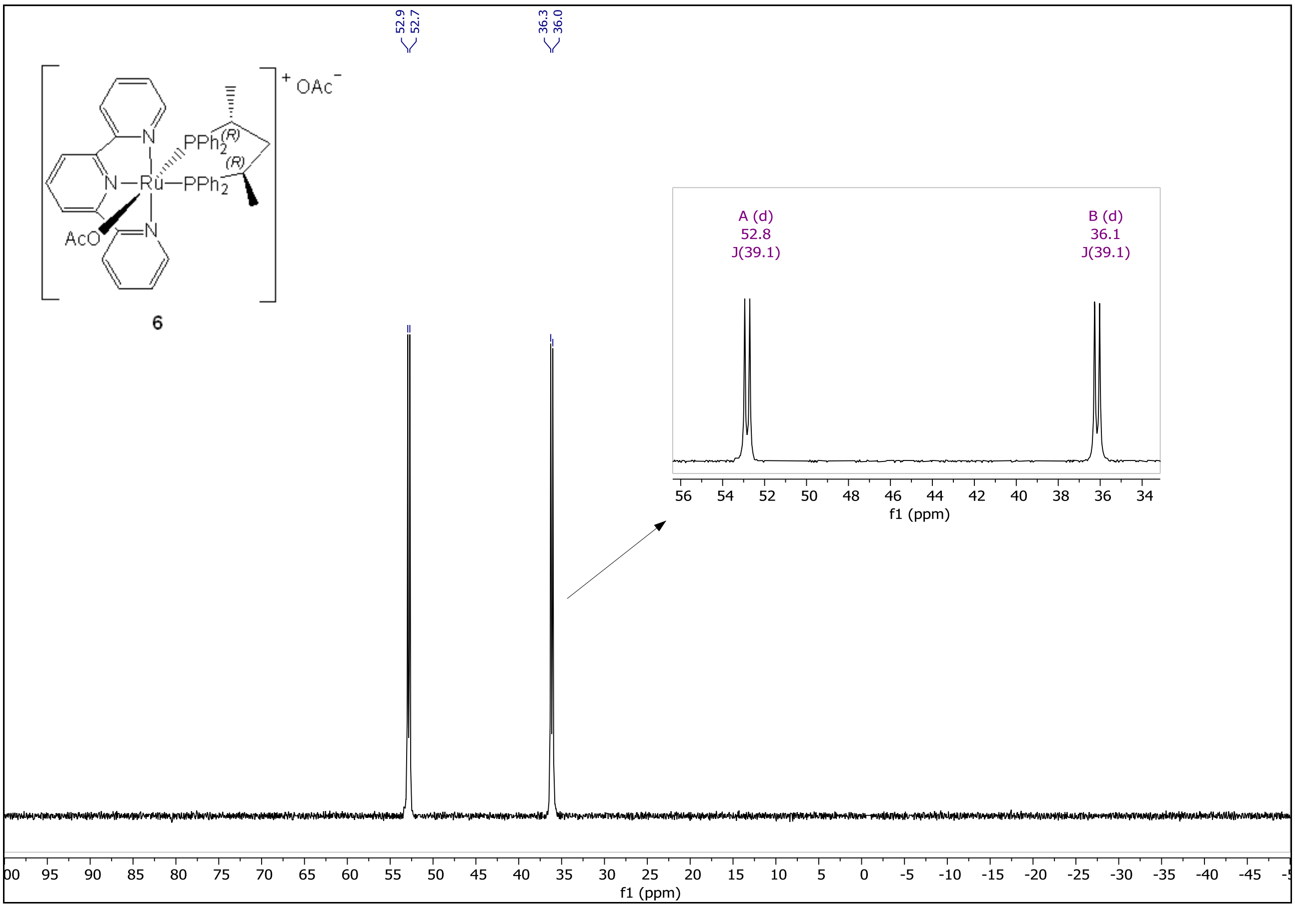

Figure S24. $^{31}P\{^{1}H\}$ NMR spectrum (162.0 MHz) of [Ru(η^1-OAc)(NNN-tpy)((*R*,*R*)-Skewphos)]OAc (**6**) in CD_3OD at 25 °C.

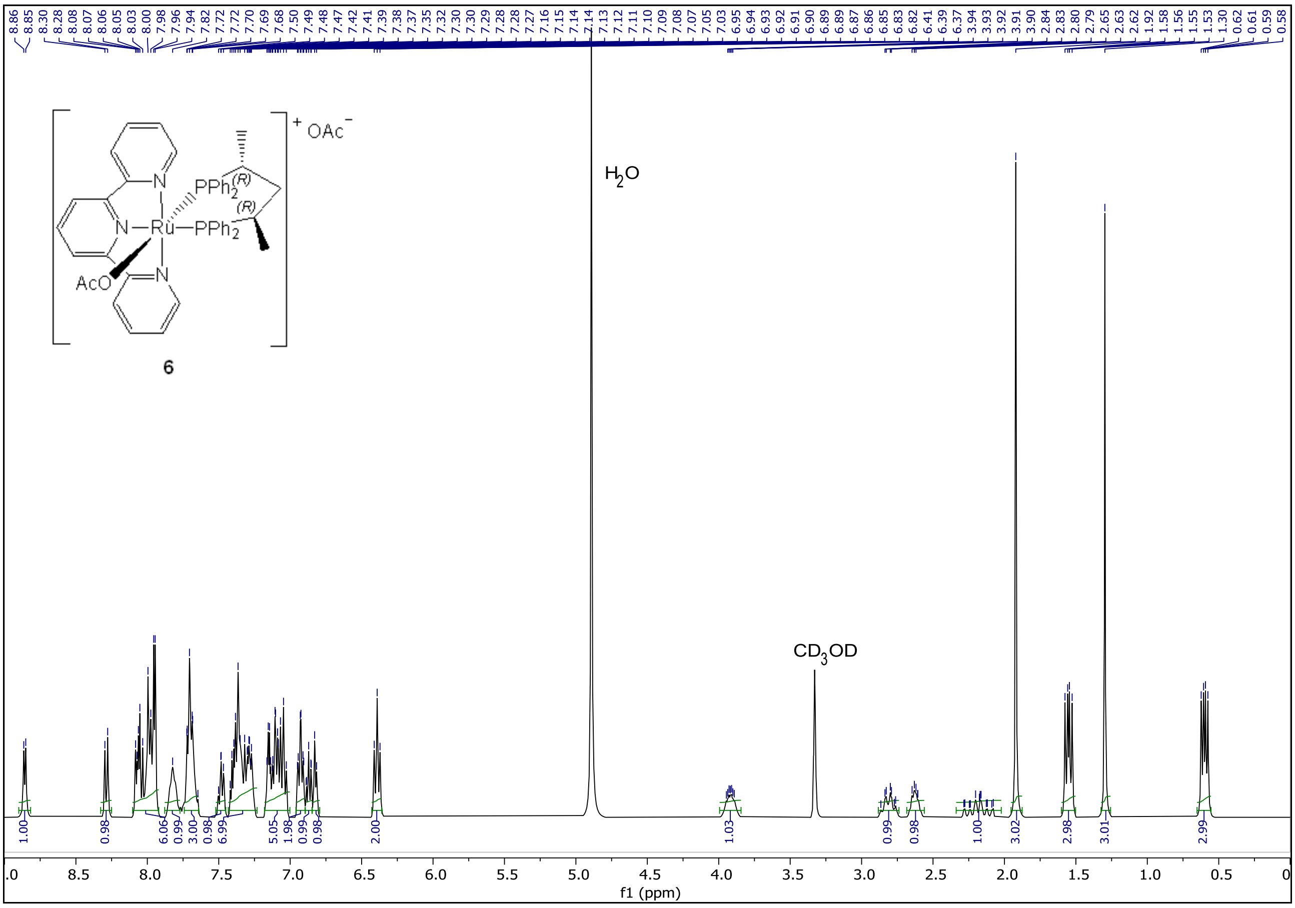

Figure S25. ^{1}H NMR spectrum (400.1 MHz) of [Ru(η^1-OAc)(NNN-tpy)((*R*,*R*)-Skewphos)]OAc (**6**) in CD_3OD at 25 °C.

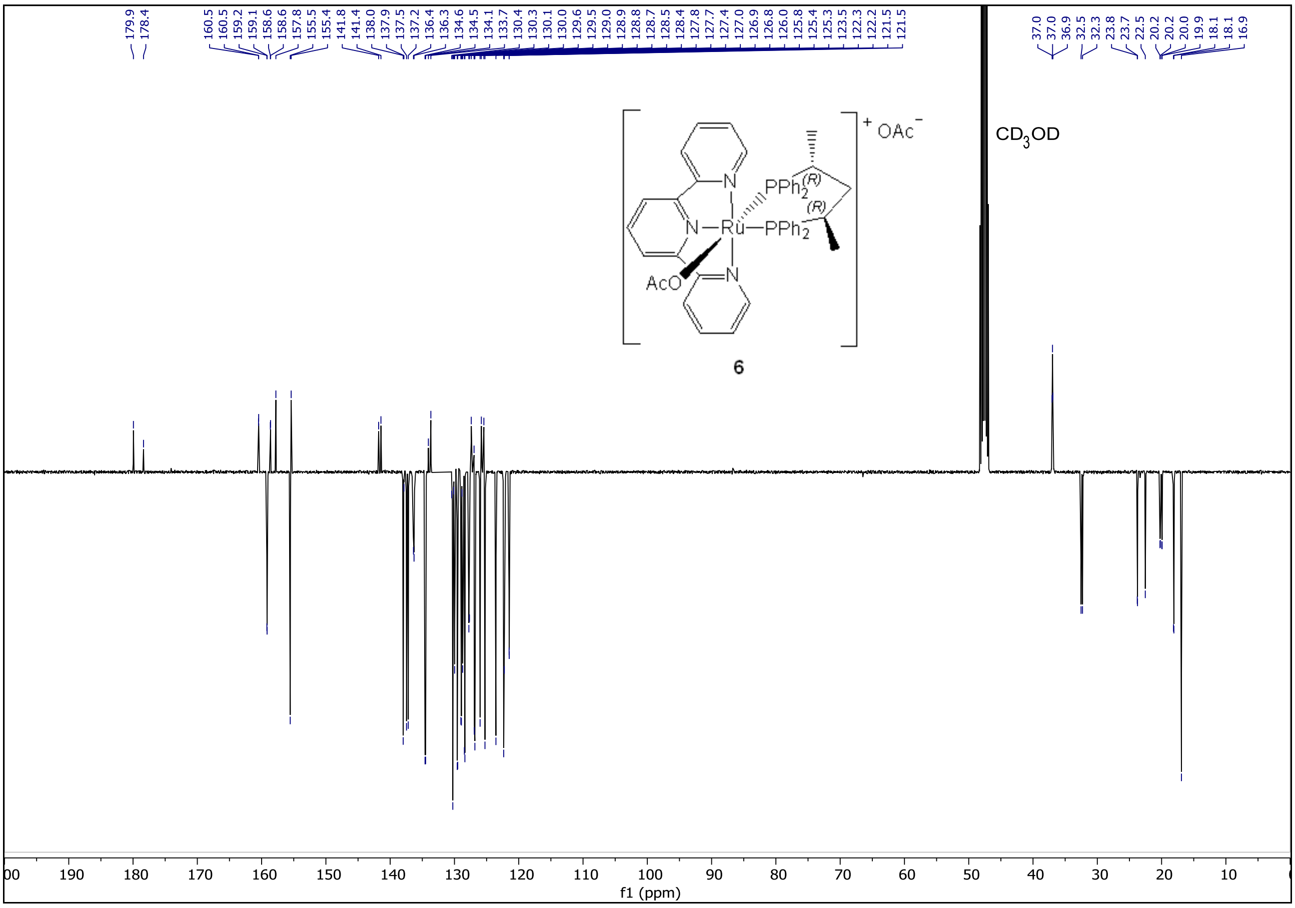

Figure S26. $^{13}C\{^{1}H\}$ DEPTQ NMR spectrum (100.6 MHz) of [Ru(η^1-OAc)(NNN-tpy)((*R*,*R*)-Skewphos)]OAc (**6**) in CD_3OD at 25 °C.

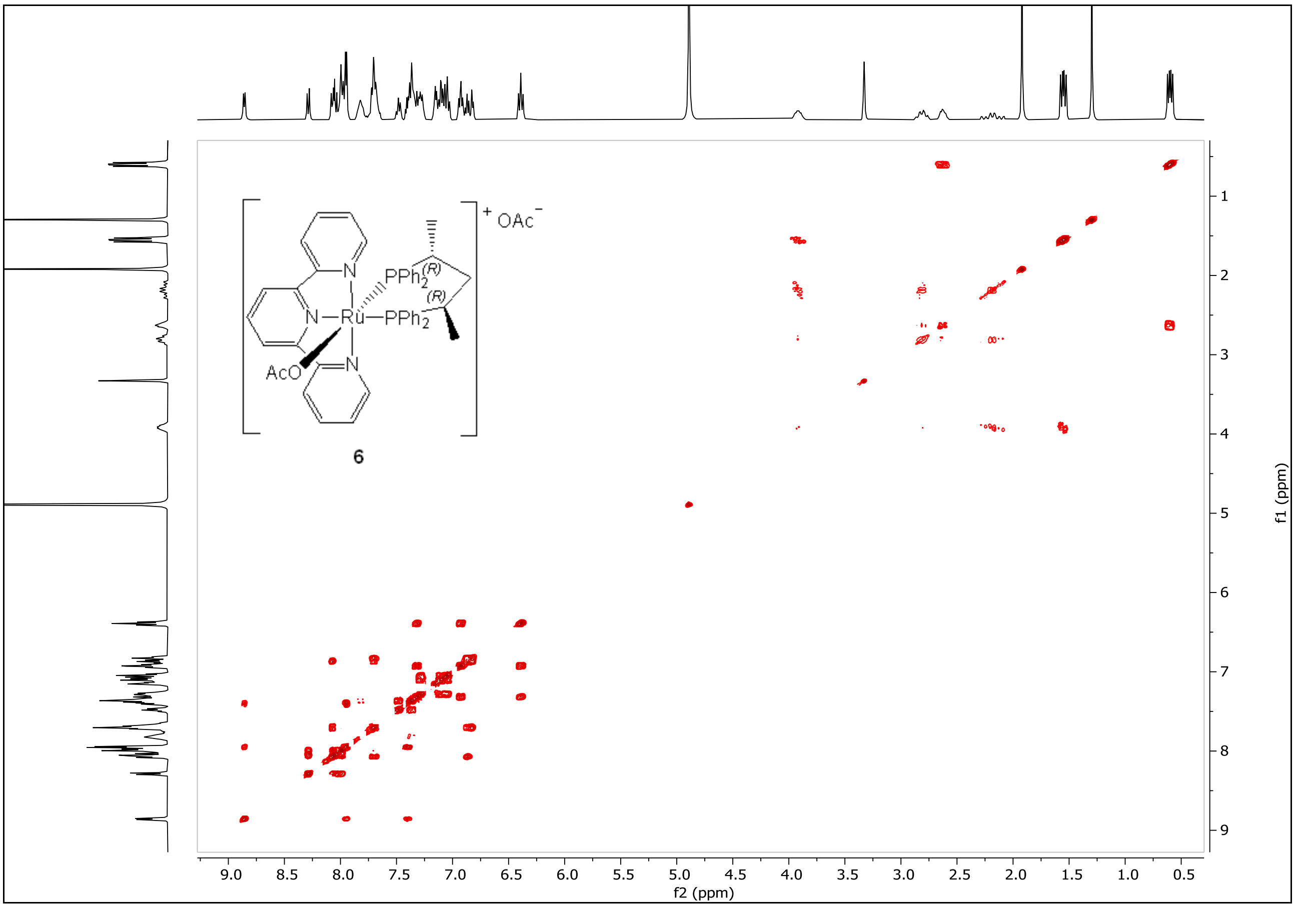

Figure S27. ^{1}H-^{1}H COSY 2D NMR spectrum of [Ru(η^{1}-OAc)(NNN-tpy)((*R*,*R*)-Skewphos)]OAc (**6**) in CD_3OD at 25 °C.

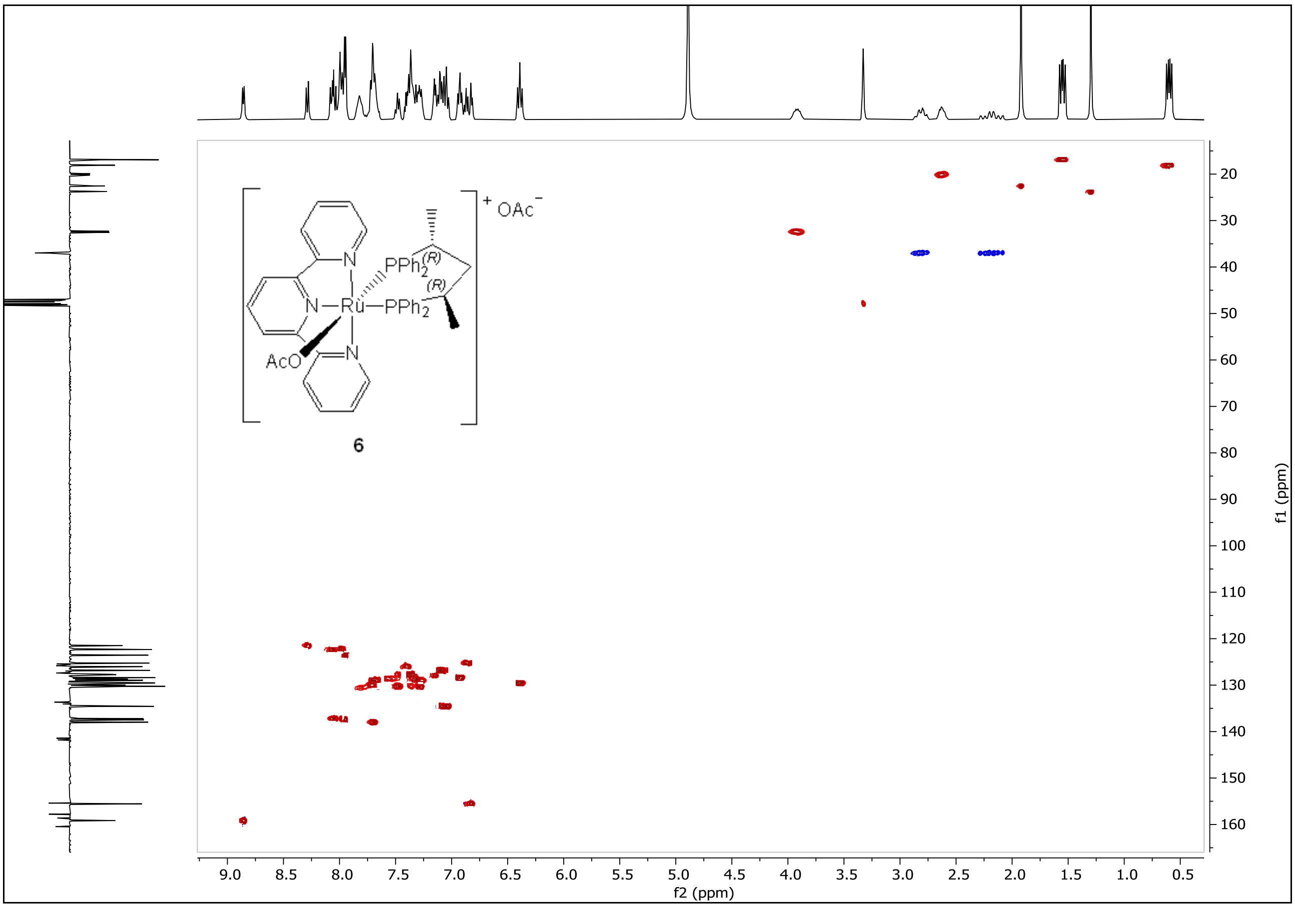

Figure S28. ^{1}H-^{13}C HSQC 2D NMR spectrum of [Ru(η^{1}-OAc)(NNN-tpy)((*R*,*R*)-Skewphos)]OAc (**6**) in CD_3OD at 25 °C.

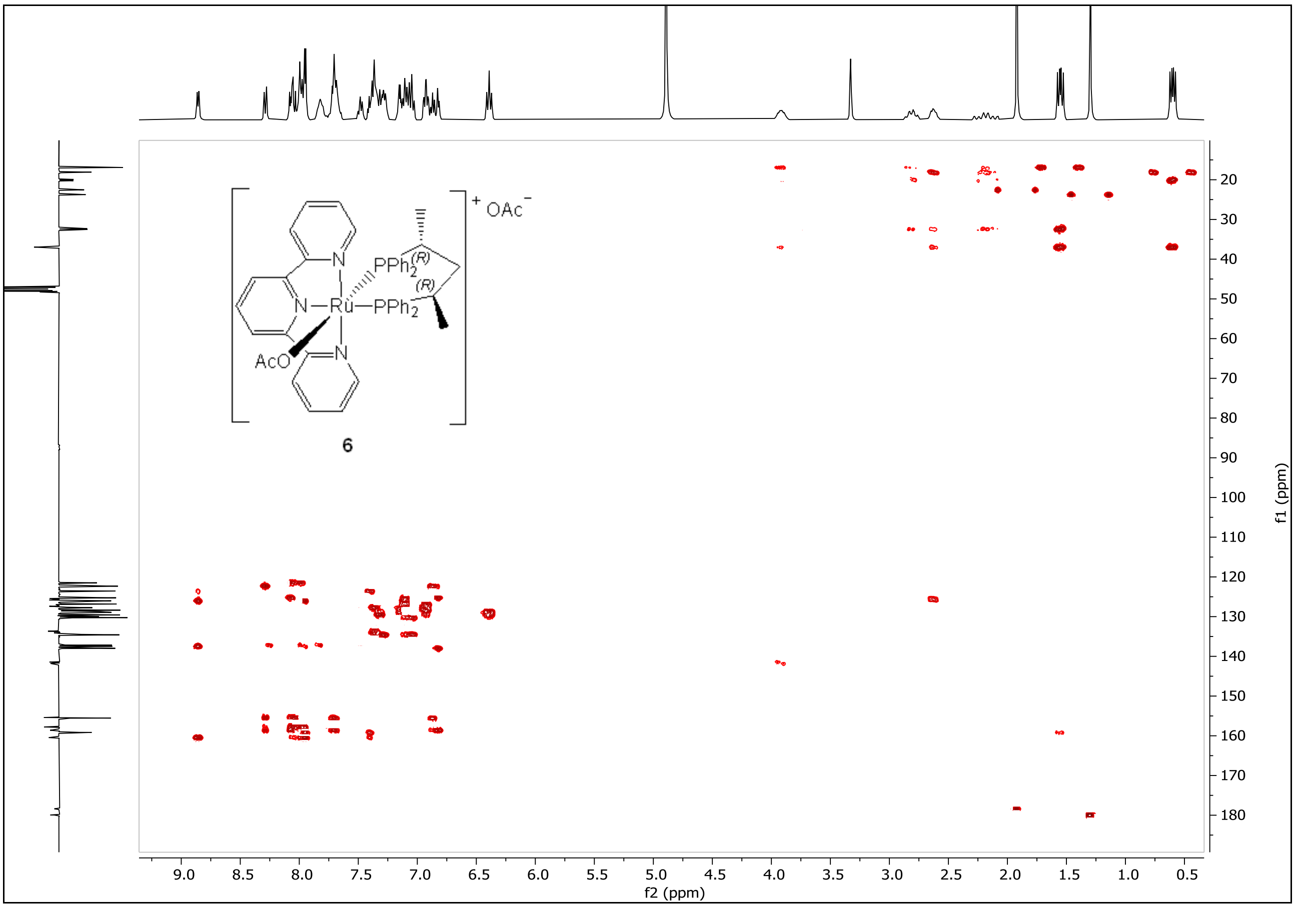

Figure S29. ^{1}H-^{13}C HMBC 2D NMR spectrum of [Ru(η^1-OAc)(NNN-tpy)((*R*,*R*)-Skewphos)]OAc (**6**) in CD_3OD at 25 °C.

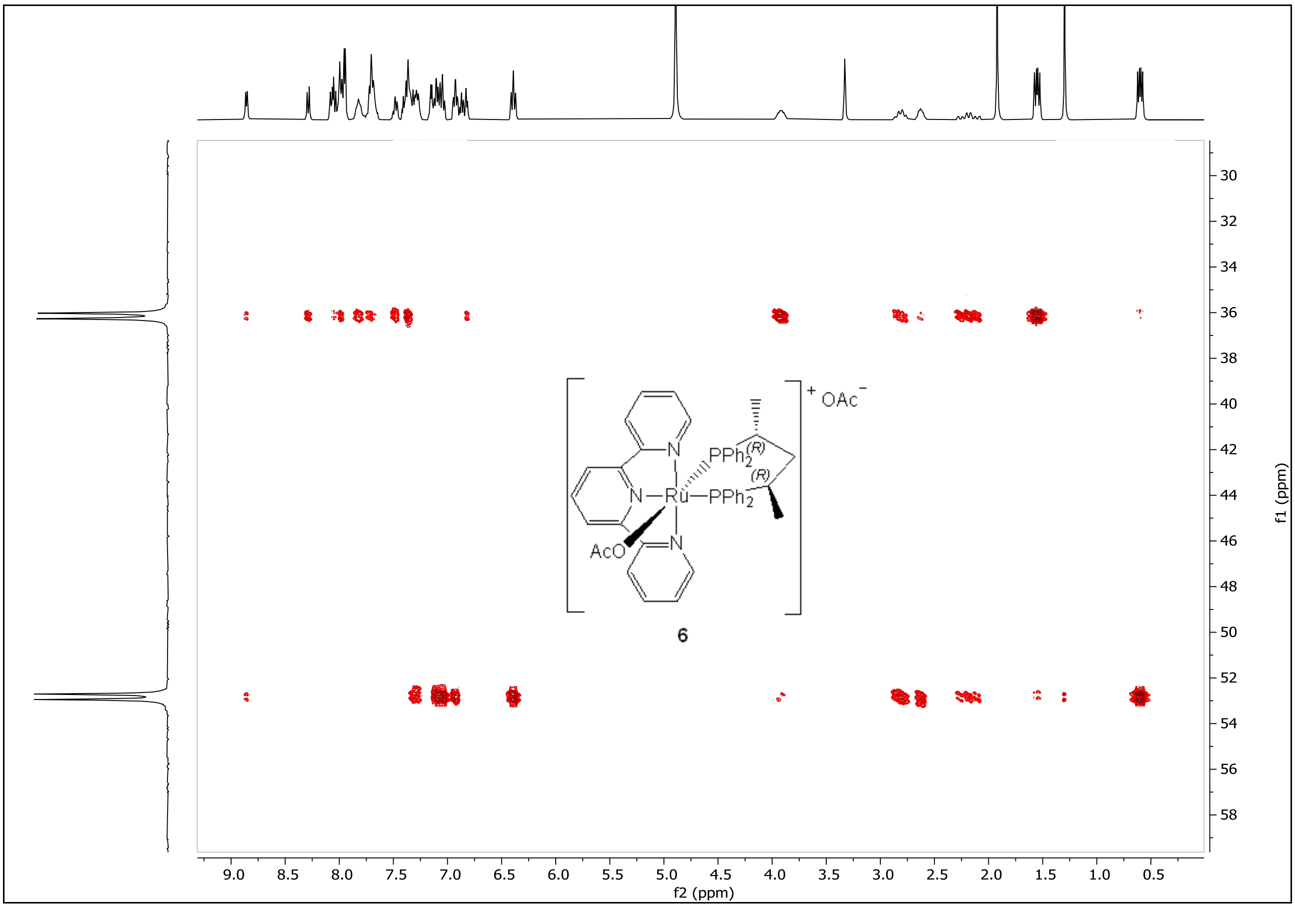

Figure S30. ^{1}H-^{31}P HMBC 2D NMR spectrum of [Ru(η^{1}-OAc)(NNN-tpy)((*R*,*R*)-Skewphos)]OAc (**6**) in CD_3OD at 25 °C.

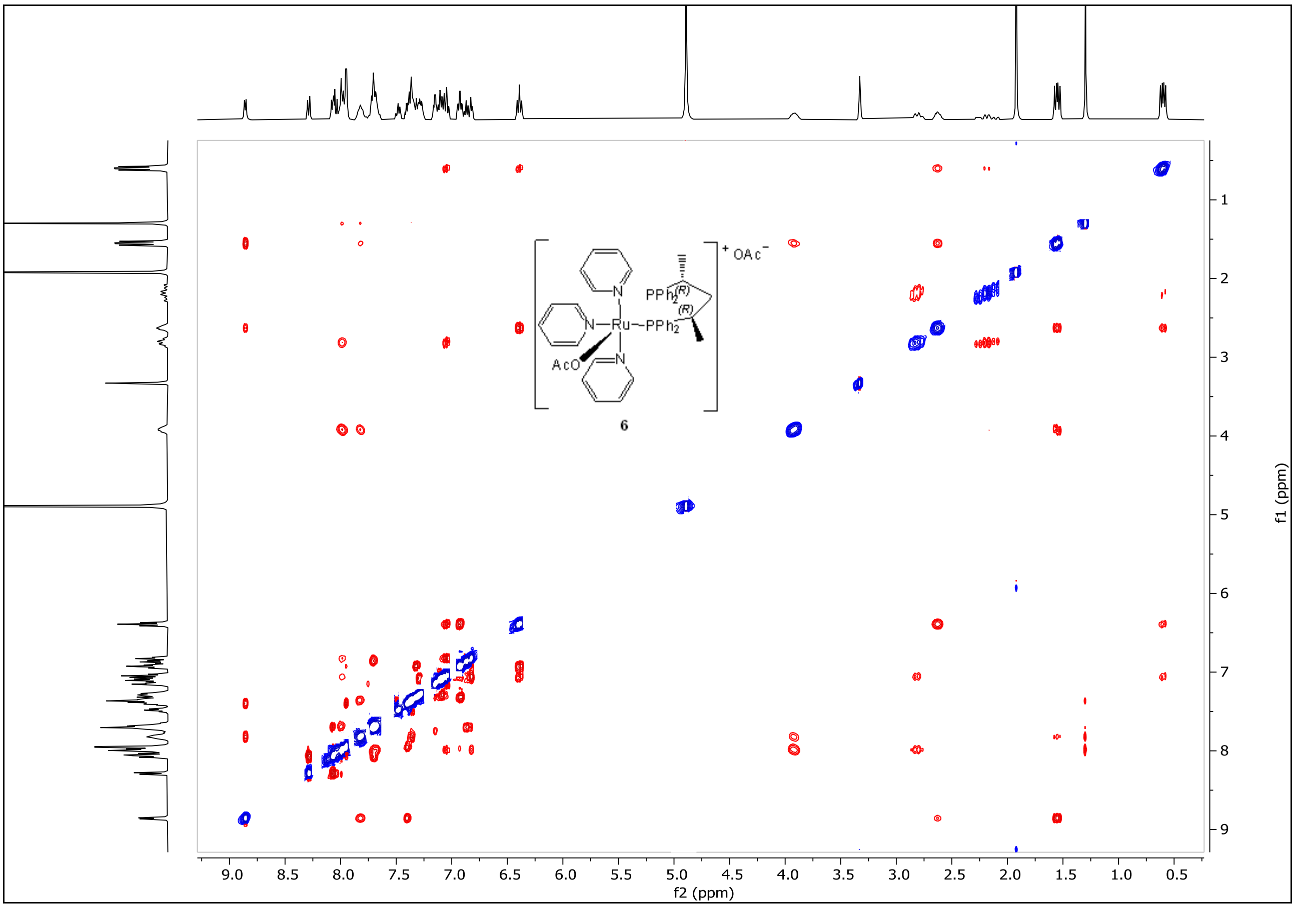

Figure S31. ^{1}H-^{1}H NOESY 2D NMR spectrum of [Ru(η^{1}-OAc)(NNN-tpy)((*R*,*R*)-Skewphos)]OAc (**6**) in CD_3OD at 25 °C.

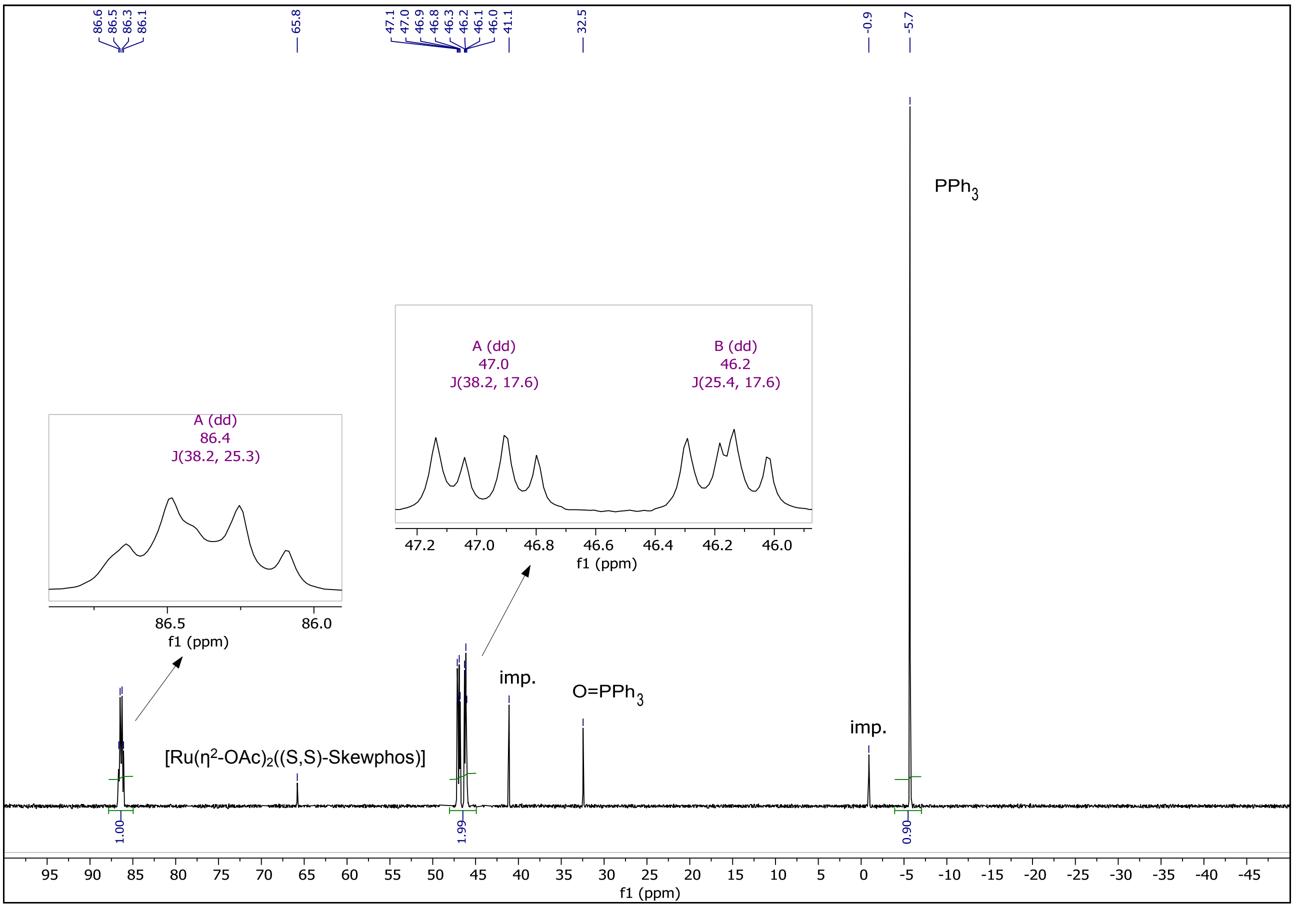

Figure S32. Evidence of the formation of the [Ru(OAc)$_2$((*S*,*S*)-Skewphos)(PPh$_3$)] intermediate in the reaction of (*S*,*S*)-Skewphos with [Ru(η^2-OAc)$_2$(PPh$_3$)$_2$] in methanol at reflux in the ^{31}P{^{1}H} NMR spectrum (162.0 MHz) with CD$_3$OD as lock at 25 °C.

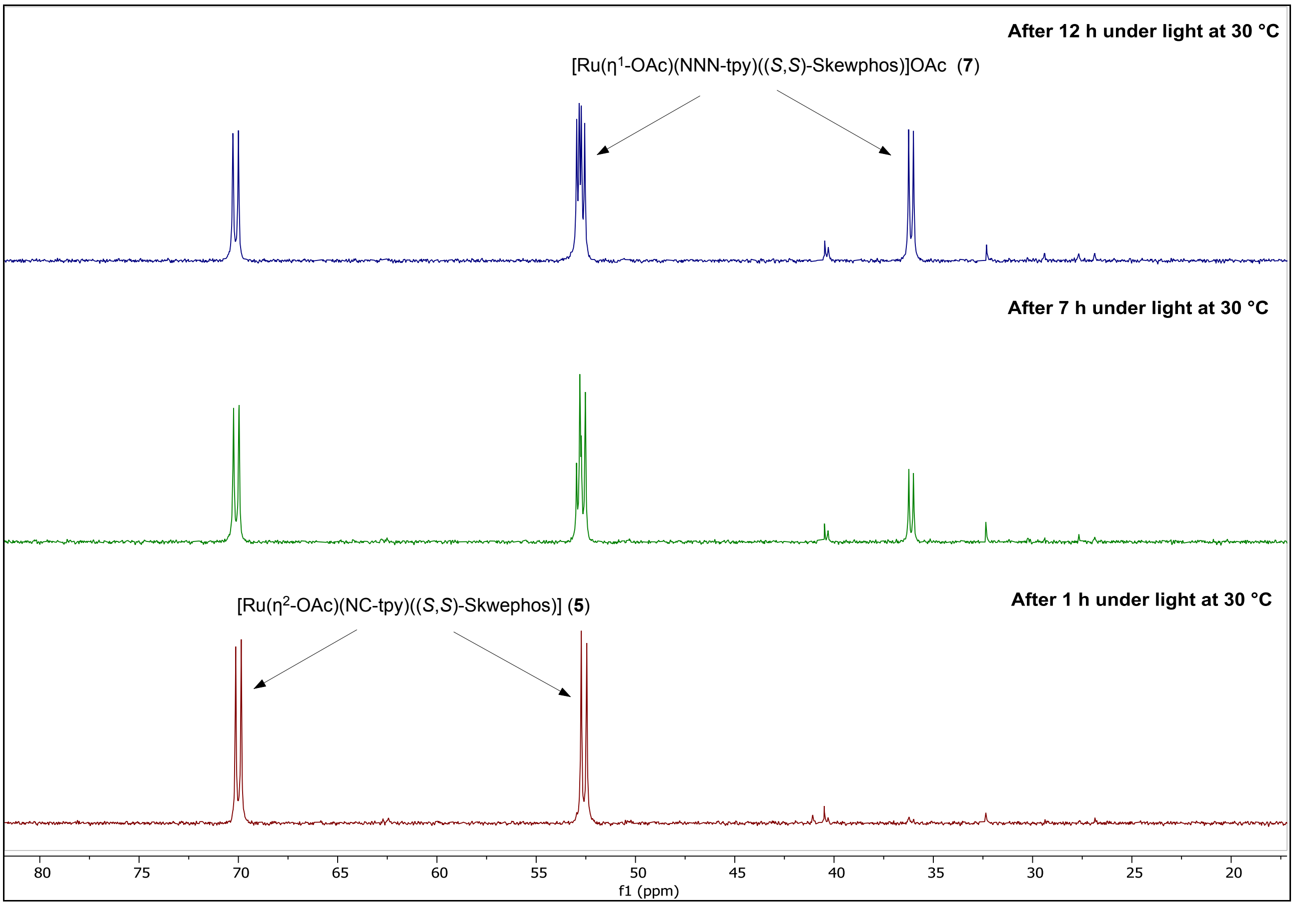

Figure S33. Conversion of complex [Ru(η^2-OAc)(NC-tpy)((*S*,*S*)-Skewphos)] (**5**) to the cationic specie [Ru(η^1-OAc)(NNN-tpy)((*S*,*S*)-Skewphos)]OAc (**7**) promoted by light with acetic acid (3 equiv) at 30 °C in the $^{31}P\{^1H\}$ NMR spectra (162.0 MHz) in CD_3OD (recorded at 25 °C).

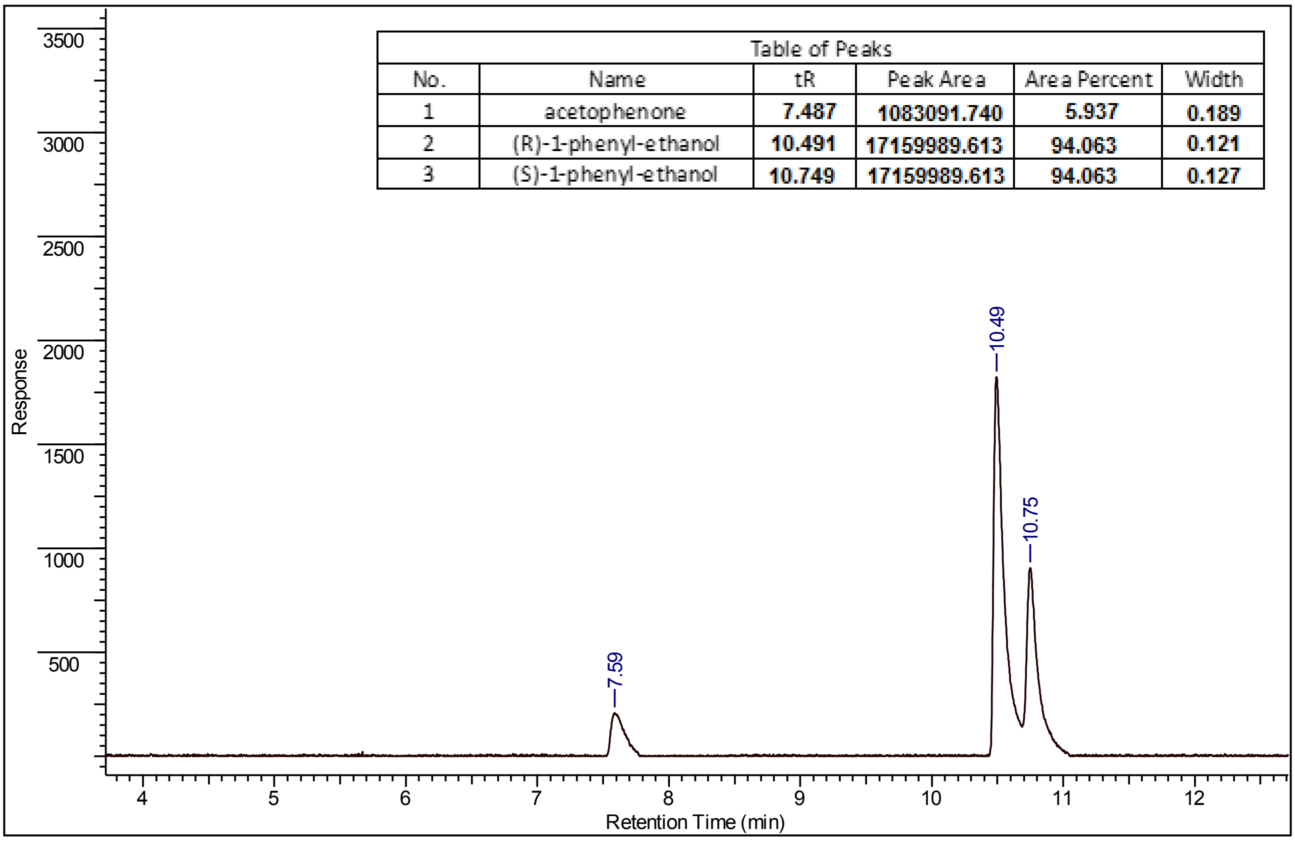

Table of Peaks					
No.	Name	tR	Peak Area	Area Percent	Width
1	acetophenone	7.487	1083091.740	5.937	0.189
2	(R)-1-phenyl-ethanol	10.491	17159989.613	94.063	0.121
3	(S)-1-phenyl-ethanol	10.749	17159989.613	94.063	0.127

Figure S34. GC-FID chromatogram of the reaction mixture of the enantioselective photocatalytic TH of acetophenone in 2-propanol/MeOH 1:1 (v/v) at 30 °C and NaO*i*Pr 2 mol% promoted by complex **7** at S/C 1000 after 28 h of overall irradiation. GC analyses were performed with a Varian CP-3380 gas chromatograph equipped with a 25 m length MEGADEX-ETTBDMS-β chiral column with hydrogen (5 psi) as the carrier gas and flame ionization detector (FID). The injector and detector temperature was 250 °C, with initial T = 95 °C ramped to 140 °C at 3 °C/min for a total of 15 min of analysis.

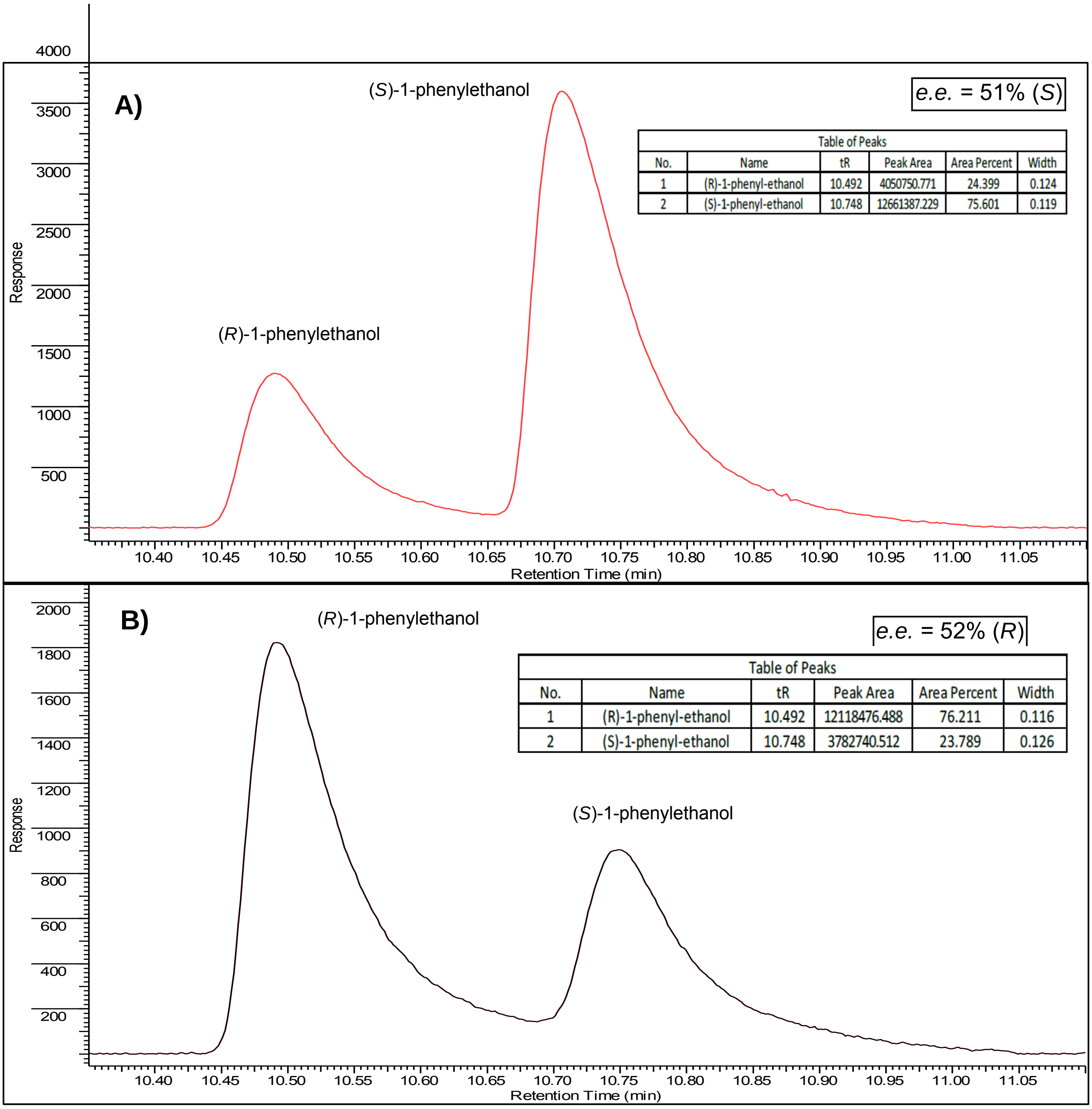

Figure S35. Comparison between the GC-FID chromatograms of the enantioselective photocatalytic TH of acetophenone in 2-propanol/MeOH 1:1 (v/v) with NaO*i*Pr 2 mol% at 30 °C promoted by complex **6** (A) and **7** (B) at S/C 1000.

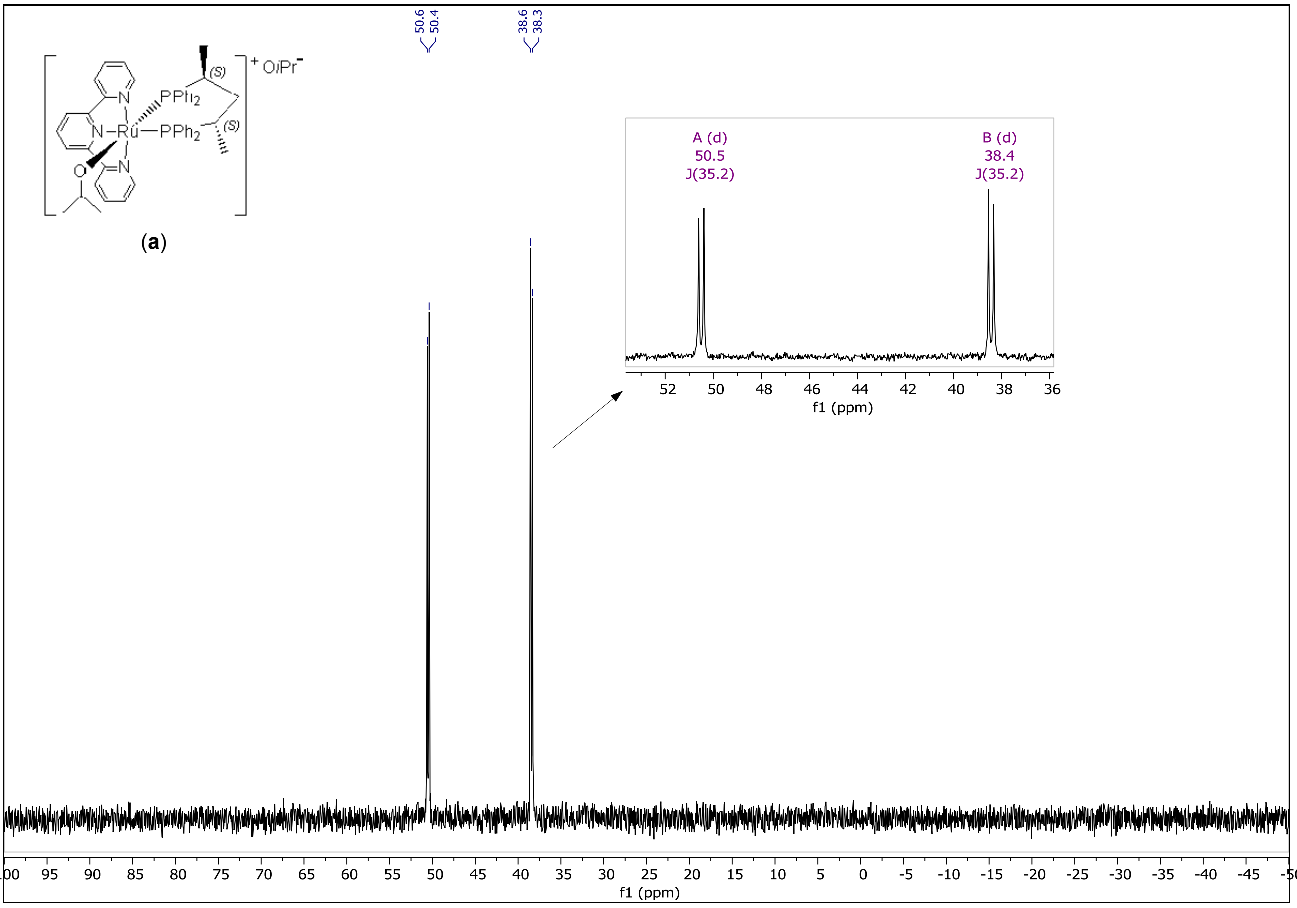

Figure S36. $^{31}P\{^1H\}$ NMR spectrum (162.0 MHz) of [Ru(O*i*Pr)(NNN-tpy)((*S*,*S*)-Skewphos)](O*i*Pr) (**a**) in 2-propanol-d^8 at 25 °C, obtained after 1 h of visible light irradiation from complex **7** in presence of NaO*i*Pr (3 equiv).

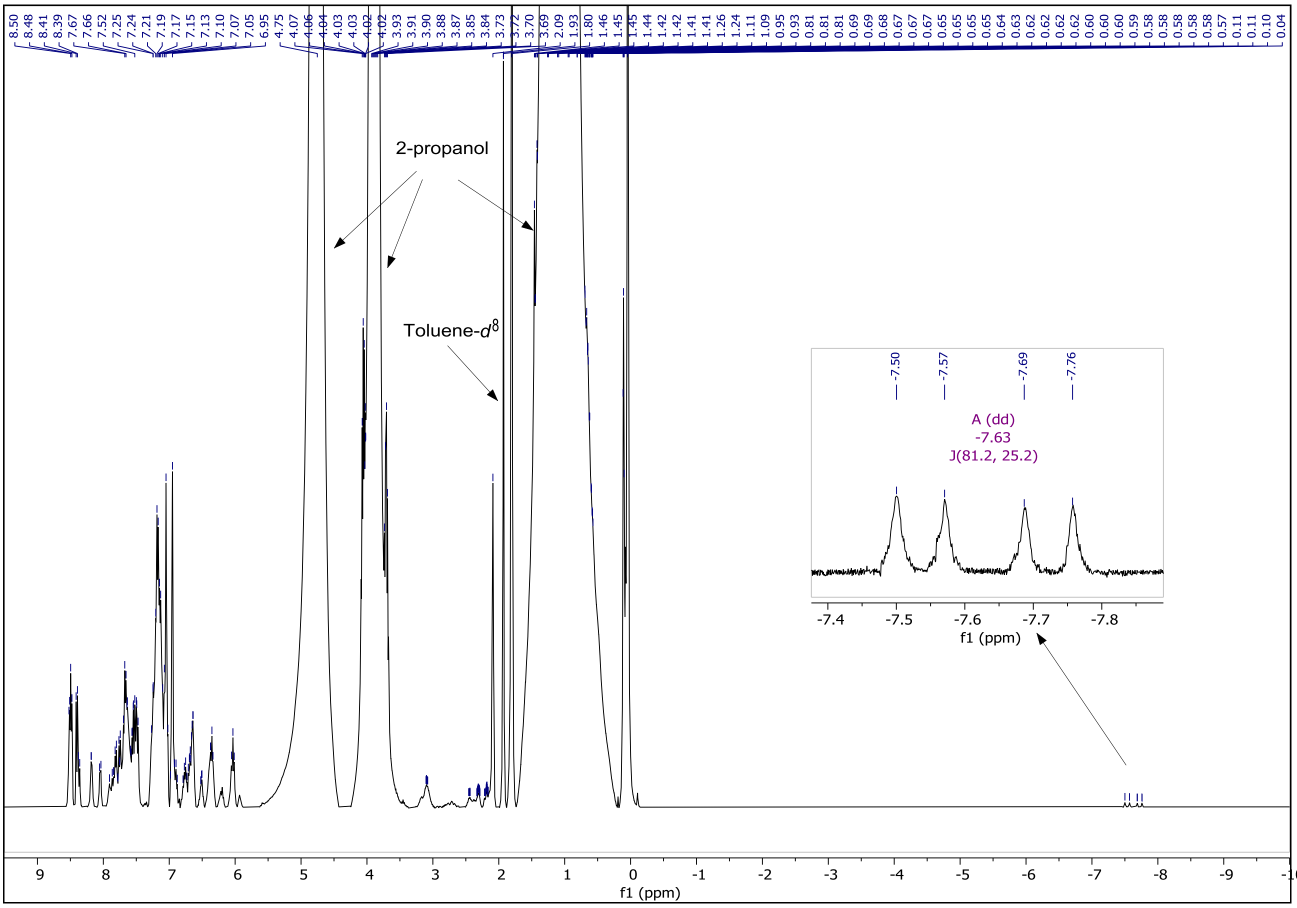

Figure S37. ^{1}H NMR spectrum (400.1 MHz) with evidences of the formation of [RuH(NNN-tpy)((*S*,*S*)-Skewphos)](O*i*Pr) (**b**) in 2-propanol/toluene-d^8 1:1 (v/v) at 25 °C (in mixture with uncharacterized compounds), obtained after 6 h of visible light irradiation from complex **5** in presence of NaO*i*Pr (3 equiv).

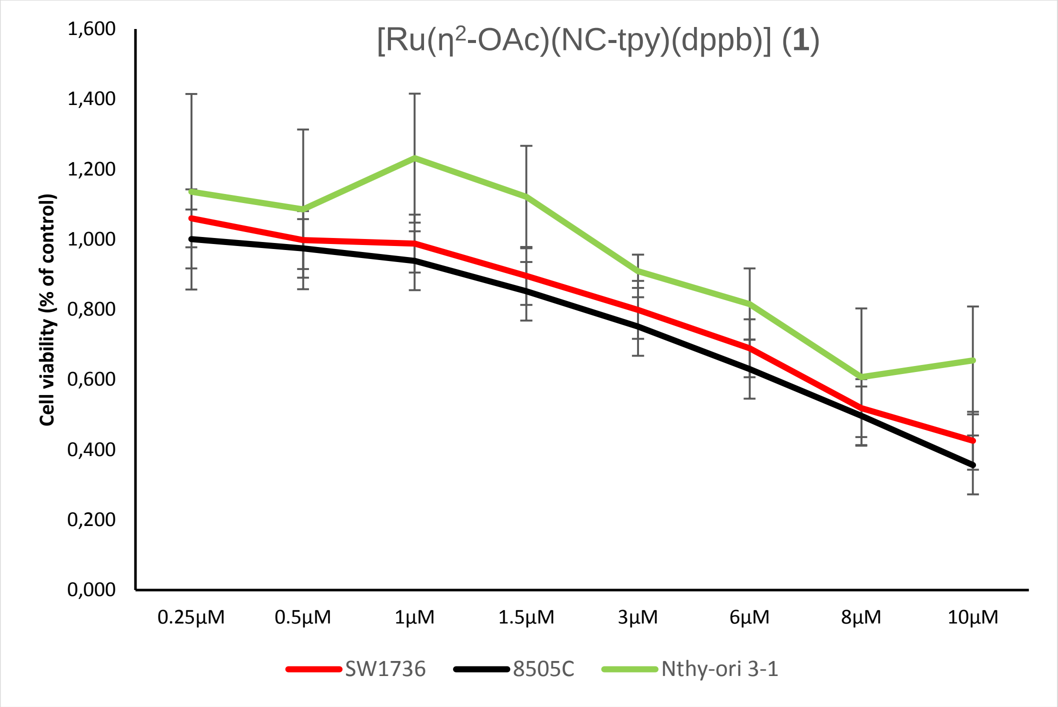

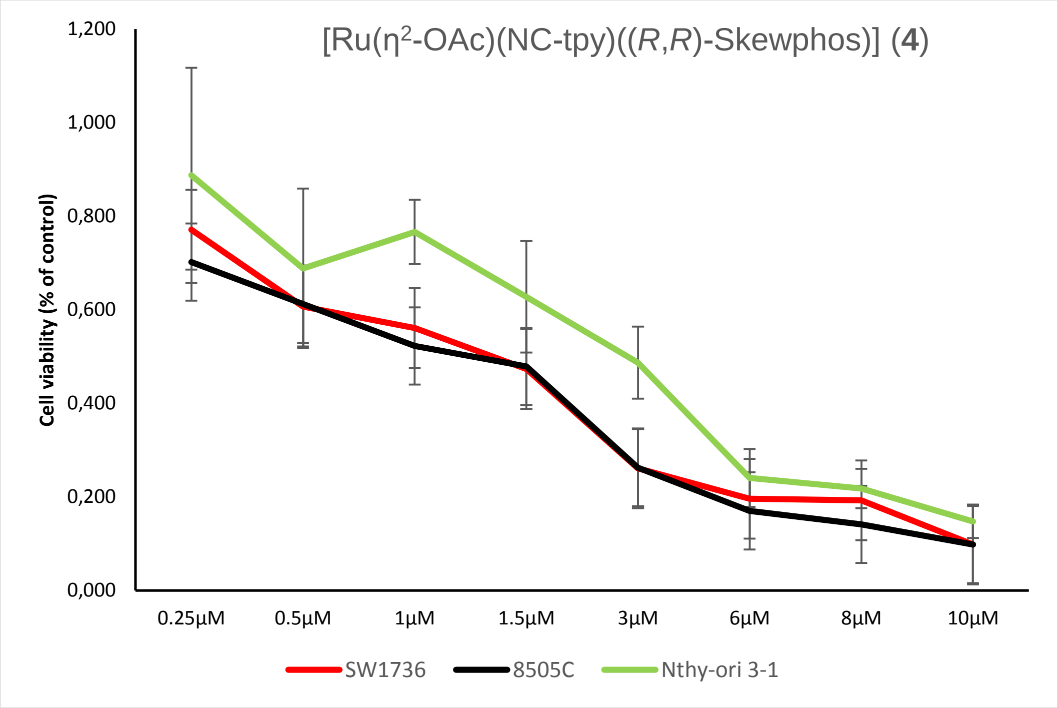

Figure S38. Effect of complexes **1** and **4** on ATC cell viability in SW1736 (red), 8505C (black) and Nthy-ori 3-1 (light green) cells. Cell viability was evaluated by using the MTT assay and expressed as the percentage of control (cells treated with DMSO alone). Each point represents the mean value of six-fold determinations. P-values obtained by statistical analysis (student's t test) are listed in the Table S1.

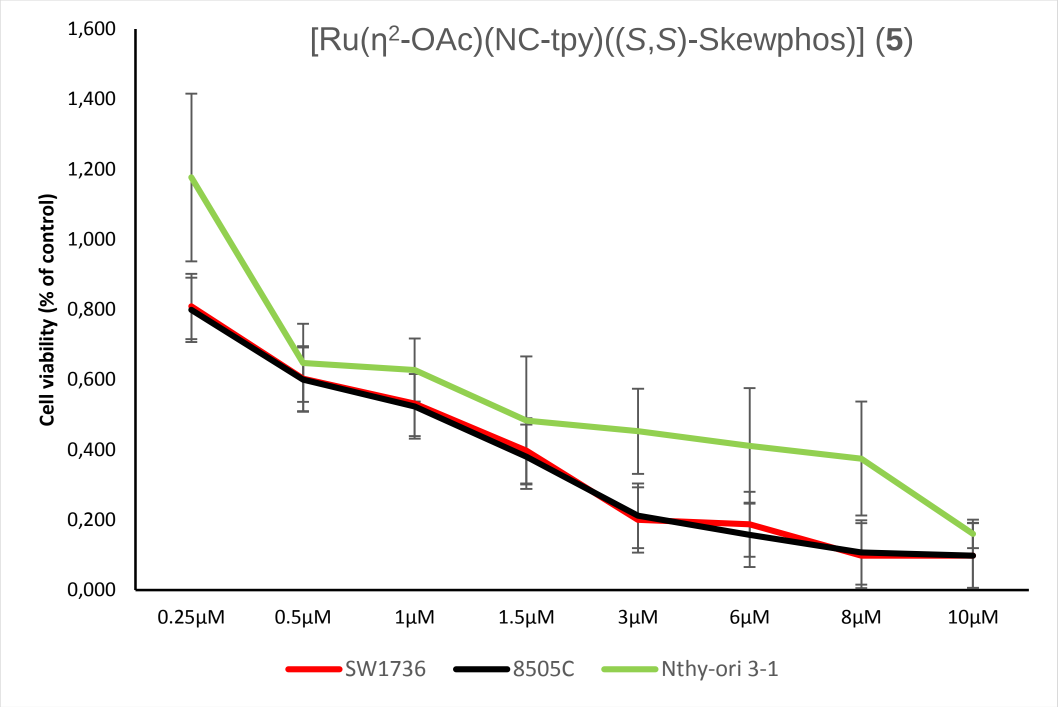

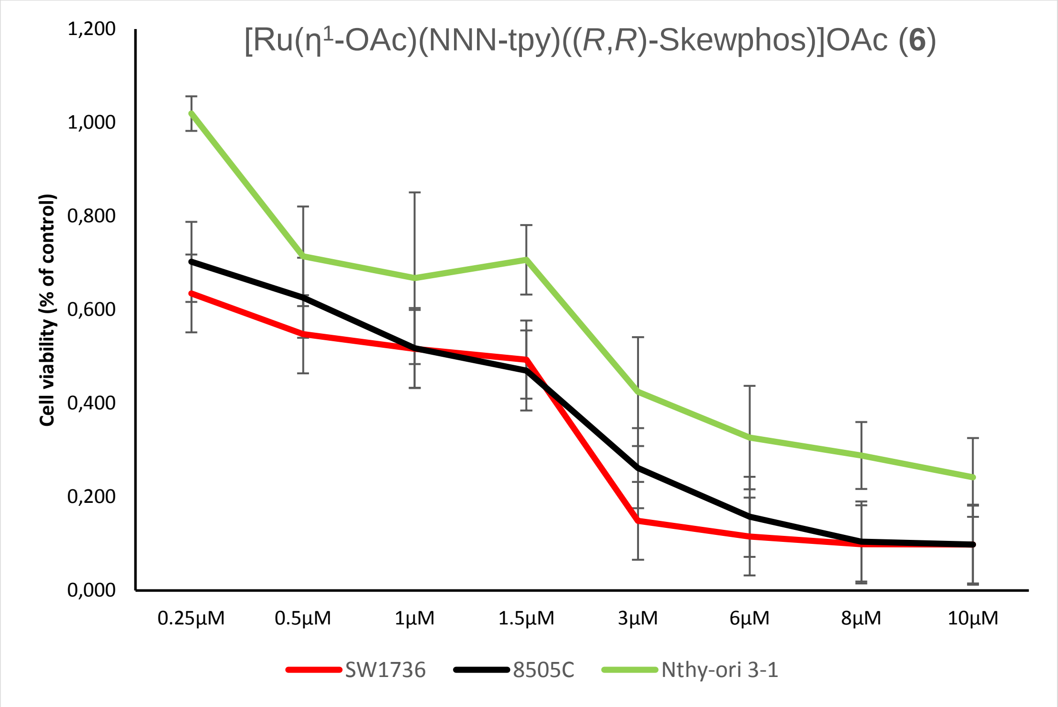

Figure S39. Effect of complexes **5** and **6** on ATC cell viability in SW1736 (red), 8505C (black) and Nthy-ori 3-1 (light green) cells. Cell viability was evaluated by using the MTT assay and expressed as the percentage of control (cells treated with DMSO alone). Each point represents the mean value of six-fold determinations. P-values obtained by statistical analysis (student's t test) are listed in the Table S1.

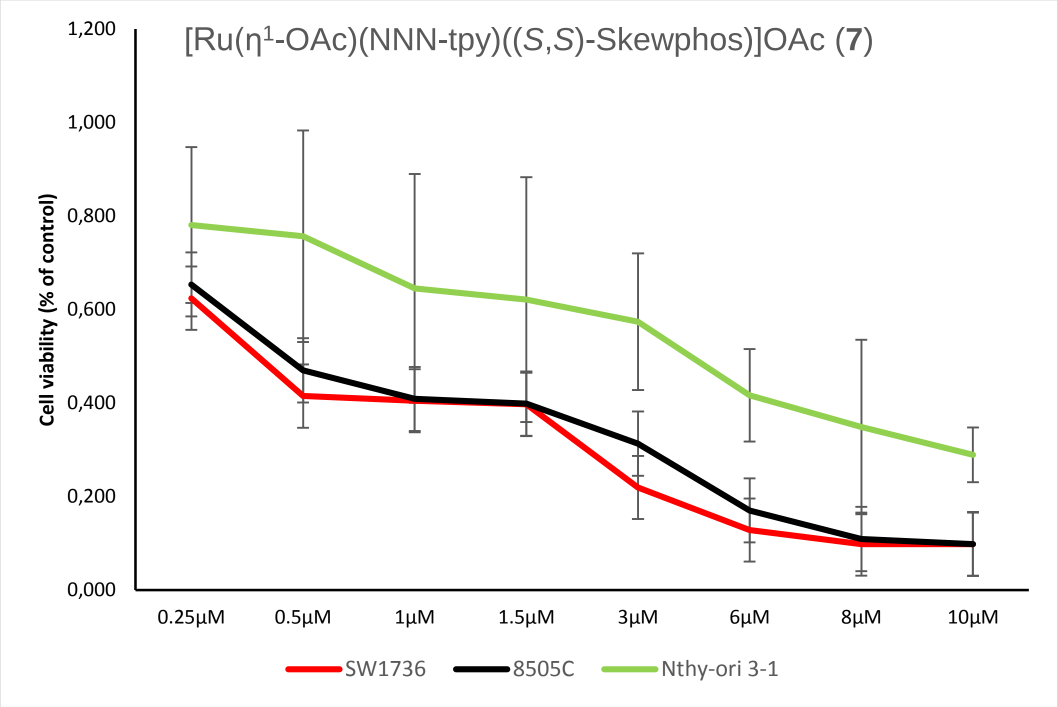

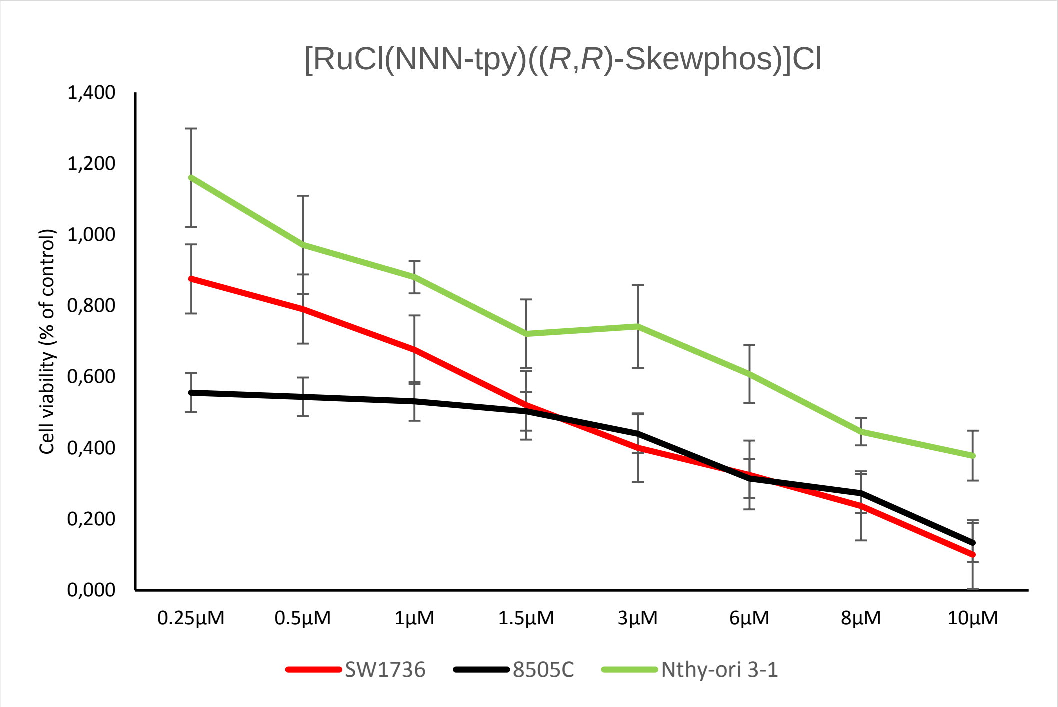

Figure S40. Effect of complexes **7** and [RuCl(NNN-tpy)((*R*,*R*)-Skewphos)]Cl on ATC cell viability in SW1736 (red), 8505C (black) and Nthy-ori 3-1 (light green) cells. Cell viability was evaluated by using the MTT assay and expressed as the percentage of control (cells treated with DMSO alone). Each point represents the mean value of six-fold determinations. P-values obtained by statistical analysis (student's t test) are listed in the Table S1.

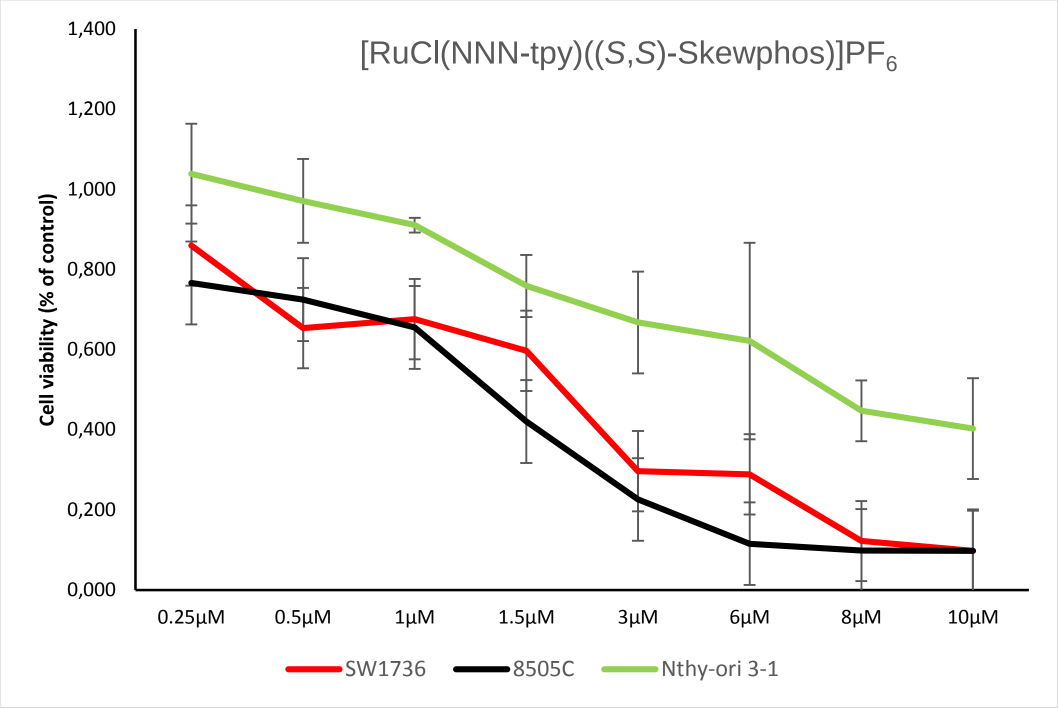

Figure S41. Effect of complex [RuCl(NNN-tpy)((*S*,*S*)-Skewphos)]PF_6 on ATC cell viability in SW1736 (red), 8505C (black) and Nthy-ori 3-1 (light green) cells. Cell viability was evaluated by using the MTT assay and expressed as the percentage of control (cells treated with DMSO alone). Each point represents the mean value of six-fold determinations. P-values obtained by statistical analysis (student's t test) are listed in the Table S1.

	[RuCl(NNN-tpy)((*R,R*)-Skewphos)]Cl			[RuCl(NNN-tpy)((*S,S*)-Skewphos)]PF_6			[Ru(η^1-OAc)(NNN-tpy)((*R,R*)-Skewphos)]OAc (**6**)			[Ru(η^2-OAc)(NC-tpy)(dppb)] (**1**)			[Ru(η^2-OAc)(NC-tpy)((*R,R*)-Skewphos)] (**4**)			[Ru(η^1-OAc)(NNN-tpy)((*S,S*)-Skewphos)]OAc (**7**)			[Ru(η^2-OAc)(NC-tpy)((*S,S*)-Skewphos)] (**5**)		
**complex dose**	SW1736	8505C	Nthy-ori 3-1	SW1736	8505C	Nthy-ori 3-1	SW1736	8505C	Nthy-ori 3-1	SW1736	8505C	Nthy-ori 3-1	SW1736	8505C	Nthy-ori 3-1	SW1736	8505C	Nthy-ori 3-1	SW1736	8505C	Nthy-ori 3-1
**0.25μM**	*	*	NS	***	*	NS	*	***	NS	NS	NS	NS	*	*	NS	*	*	*	NS	NS	NS
**0.5μM**	NS	*	NS	*	*	NS	***	*	***	NS	NS	NS	****	****	*	****	****	NS	NS	NS	***
**1μM**	*	*	***	*	*	****	***	***	*	NS	NS	*	*	***	***	****	***	*	*	NS	****
**1.5μM**	****	*	***	****	*	****	***	***	****	*	NS	NS	***	****	***	****	***	*	****	*	*
**3μM**	***	*	*	****	****	***	****	****	****	NS	NS	*	****	****	****	****	****	***	****	****	****
**6μM**	***	*	****	****	****	*	****	****	****	*	*	*	****	****	****	****	****	****	****	****	****
**8μM**	***	*	****	****	****	****	****	****	****	****	***	*	****	****	****	****	****	****	****	****	****
**10μM**	****	****	****	****	****	****	****	****	****	****	***	*	****	****	****	****	****	****	****	****	****

Table S1. Statistical analysis for tested complexes. For each dose of each complex, the p-value in each of the two cell lines is listed. NS: not significant, * $p<0.05$, ** $p<0.01$, *** $p<0.001$, **** $p<0.0001$.

Single Crystal X-Ray Structure Determination of Compound 1 (CCDC 2302606).

General Data

Data were collected on a Bruker D8 Venture single crystal x-ray diffractometer equipped with a CPAD detector (Bruker Photon II), an IMS microsource with MoK_{α} radiation (λ = 0.71073 Å) and a Helios optic using the APEX3 software package.[83] The measurement was performed on a single crystal coated with perfluorinated ether which was fixed on top of a kapton micro sampler and frozen under a stream of cold nitrogen. A matrix scan was used to determine the initial lattice parameters. Reflections were corrected for Lorentz and polarisation effects, scan speed, and background using SAINT.[84] Absorption correction, including odd and even ordered spherical harmonics was performed using SADABS.[85] Space group assignment was based upon systematic absences, E statistics, and successful refinement of the structure. The structure was solved using SHELXT with the aid of successive difference Fourier maps, and was refined against all data using SHELXL in conjunction with SHELXLE.[86-88] Hydrogen atoms were calculated in ideal positions as follows: Methyl hydrogen atoms were refined as part of rigid rotating groups, with a C–H distance of 0.98 Å and $U_{iso}(H) = 1.5\cdot U_{eq}(C)$. Non-methyl H atoms were placed in calculated positions and refined using a riding model with methylene, aromatic, and other C–H distances of 0.99 Å, 0.95 Å and 1.00 Å, respectively, and $U_{iso}(H) = 1.2\cdot U_{eq}(C)$. Non-hydrogen atoms were refined with anisotropic displacement parameters. Full-matrix least-squares refinements were carried out by minimizing $\Sigma w(F_o^2 - F_c^2)^2$ with the SHELXL weighting scheme.[86] Neutral atom scattering factors for all atoms and anomalous dispersion corrections for the non-hydrogen atoms were taken from *International Tables for Crystallography*.[89] Images of the crystal structure were generated with Mercury.[90] CCDC 2302606 contains the supplementary crystallographic data for this paper. These data are provided free of charge by The Cambridge Crystallographic Data Centre.

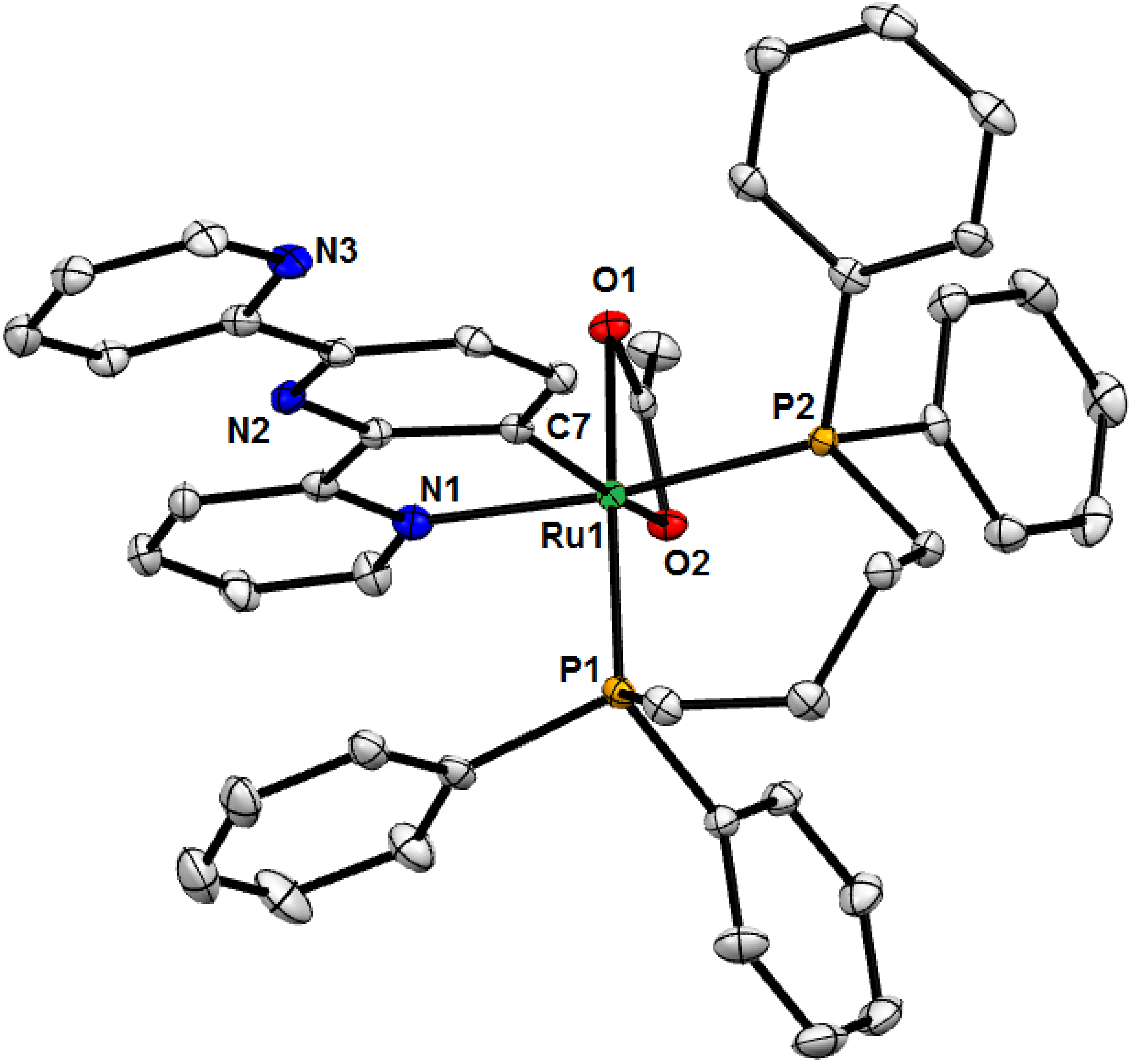

Figure S42. ORTEP style plot of compound **1** (one out of two independent molecules) in the solid state (CCDC 2302606) as determined by single crystal X-ray diffraction. Ellipsoids are displayed at the 50% probability level. Hydrogen atoms and co-crystalized solvent molecules are omitted for clarity.

Single Crystal X-Ray Structure Determination of Compound 1 (CCDC 2302606).

Detailed Crystallographic Data.

Diffractometer operator:	C. Jandl
Scanspeed	2-20 s per frame
dx	90 mm
Frames:	2785 measured in 10 XYZ data sets
phi-scans with delta phi	0.5
omega-scans with delta omega	0.5
shutterless mode	

Crystal Data:

Chemical formula: $[C_{45}H_{41}N_3O_2P_2Ru]\cdot 1/2(C_7H_{16})$		Density (calculated) = 1.396 g/cm^3
Formula weight 868.91		Absorption coefficient = 0.50 mm^{-1}
Monoclinic, *P* 1 21/n 1		Mo *K*α radiation, λ = 0.71073 Å
a = 13.196 (9) Å	α = 90°	*T* = 100(2) K
b = 44.86 (3) Å	β = 101.096(16)°	Yellow fragment
c = 14.234 (10) Å	γ = 90°	0.086 × 0.125 × 0.138 mm
V = 8269.(10) Å^3		
Z = 8		
F(000) = 3608		

Data collection:

Bruker D8 Venture Duo IMS diffractometer	15129 independent reflections
Radiation source: IMS microsource, Mo	12749 reflections with $I > 2\sigma(F^2)$
Helios optic monochromator	R_{int} = 0.1777
Theta range for data collection	θ_{max} = 25.38°, θ_{min} = 1.93°

Index ranges	-15<=h<=15, -54<=k<=54, -17<=l<=17
Absorption correction	Multi-Scan, *SADABS* 2016/2, Bruker
Max. and min. transmission	0.9580 and 0.9340
189179 measured reflections	Coverage of independent reflections = 99.9%

Data refinement:

Refinement method	Full-matrix least-squares on F^2
Refinement program	SHELXL-2018/3 (Sheldrick, 2018)
Function minimized	Σ w(Fo^2 - Fc^2)^2
Data / restraints / parameters	15129 / 0 / 1022
Final R indices	15129 data; I > 2σ(I) R1 = 0.0472, wR2 = 0.0904<br>all data R1 = 0.0614, wR2 = 0.0947
Weighting scheme	w = 1/[$\sigma^2(Fo^2)$ + $(0.0201P)^2$ + 12.9123P]<br>where P = (Fo^2 + 2Fc^2)/3
Δ/σ_{max}	0.003
Goodness-of-fit on F^2	1.109
Largest diff. peak and hole	0.524 and -0.739 eÅ^{-3}
R.M.S. deviation from mean	0.085 eÅ^{-3}

References

83) *APEX suite of crystallographic software*, APEX 3, Version 2019-1.0, Bruker AXS Inc., Madison, Wisconsin, USA, 2019.

84) *SAINT*, Bruker AXS Inc., Version 8.40A, Madison,Wisconsin, WI, USA, 2019,

85) SADABS Bruker AXS Inc., Version 2016/2, Madison,Wisconsin, WI, USA, 2016.

86) G. M. Sheldrick, Crystal structure refinement with SHELXL. *Acta Crystallogr. Sect. A* **2015**, *71*, 3-8.

87) G. M. Sheldrick, SHELXT - Integrated space-group and crystal-structure determination. *Acta Crystallogr. Sect. C* **2015**, *71*, 3-8

88) C. B. Hübschle, G. M. Sheldrick, B. Dittrich, ShelXle: a Qt graphical user interface for SHELXL. *J. Appl. Cryst.* **2011**, *44*, 1281-1284.

89) *International Tables for Crystallography, Vol. C* (Ed.: A. J. Wilson), Kluwer Academic Publishers, Dordrecht, The Netherlands, **1992**, Tables 6.1.1.4 (pp. 500-502), 4.2.6.8 (pp. 219-222), and 4.2.4.2 (pp. 193-199).

90) C. F. Macrae, I. J. Bruno, J. A. Chisholm, P. R. Edgington, P. McCabe, E. Pidcock, L. Rodriguez-Monge, R. Taylor, J. van de Streek, P. A. Wood. Mercury CSD 2.0 - new features for the visualization and investigation of crystal structures. *J. Appl. Cryst.* **2008**, *41*, 466-470.
